# Native and Engineered Extracellular Vesicles for the Treatment of Acute Lung Injury and Acute Respiratory Distress Syndrome

**DOI:** 10.1002/smsc.202400606

**Published:** 2025-02-26

**Authors:** Zhengyan Gu, Wenjun Xue, Guanchao Mao, Zhipeng Pei, Jingjing Li, Mingxue Sun, Xinkang Zhang, Shanshan Zhang, Songling Li, Jinfeng Cen, Kai Xiao, Ying Lu, Qingqiang Xu

**Affiliations:** ^1^ Department of Pharmaceutical Sciences School of Pharmacy Naval Medical University Shanghai 200433 P. R. China; ^2^ Department of Organ Regeneration Shanghai East Hospital Tongji University School of Medicine Shanghai 200120 P. R. China; ^3^ Lab of Toxicology and Pharmacology Faculty of Naval Medicine Naval Medical University Shanghai 200433 P. R. China; ^4^ School of Medicine Tongji University Shanghai 200070 P. R. China; ^5^ School of Traditional Chinese Materia Medica Shenyang Pharmaceutical University Shenyang 110006 P. R. China; ^6^ Basic Medical Center for Pulmonary Disease Naval Medical University 800, Xiangyin Road Shanghai 200433 P. R. China

**Keywords:** acute lung injury, acute respiratory distress syndromes, engineered extracellular vesicles, extracellular vesicles

## Abstract

Extracellular vesicles (EVs) are lipid bilayer nanoparticles naturally released from cells, playing a crucial role in intercellular communication. They modulate gene expression and regulate physiological and pathological processes, including acute lung injury (ALI) and acute respiratory distress syndrome (ARDS). Research has shown that EVs contain a variety of active components, are biocompatible and small in size, and do not trigger immune rejection, making the infusion of exogenous EVs a promising therapeutic tool. With further research, engineering strategies have been proposed to enhance the clinical potential of EVs. These strategies involve modifying either donor cells that secrete EVs or the EVs themselves and can be engineered to circumvent the limitations of native EVs. In this review, an overview of the biological properties of native EVs is provided and the current therapeutic potential of native and engineered EVs in treating ALI/ARDS, along with the latest research findings, is summarized. The challenges and opportunities for clinical translation of EVs as a novel therapeutic tool are also discussed, offering new insights into the treatment of ALI/ARDS using EV engineering technology.

## Graphical abstract

Both native and engineered extracellular vesicles (EVs) are served as promising therapeutic agents for treating acute lung injury (ALI) and acute respiratory distress syndrome (ARDS). To circumvent the limitations of native EVs, various engineering strategies are adopted. The challenges and opportunities for clinical translation of EVs are also discussed, providing new perspectives for EV‐based therapy.

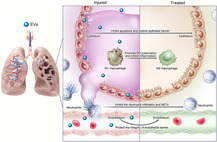

## 1 Introduction

Acute lung injury (ALI) and acute respiratory distress syndrome (ARDS) are prevalent clinical conditions characterized by respiratory distress and persistent hypoxemia.^[^ ^1^, ^2^, ^3^ ^]^ Many factors can trigger ALI either directly or indirectly, including bacterial, viral, and fungal pneumonia, as well as aspiration of stomach contents and sepsis, with the latter two being the most clinically significant factors.^[^ ^4^ ^]^ A recent international multicenter clinical cohort study reported that 10.4% of patients in intensive care units suffer from ARDS, with a mortality rate of ≈40%.^[^ ^5^ ^]^ Survivors often experience poor quality of life, characterized by exercise limitations, tracheal stenosis, muscle atrophy, and a weakened immune response.^[^ ^6^ ^]^ The COVID‐19 outbreak that began in December 2019 is highly contagious and can lead to fatal comorbidities, especially COVID‐19‐related ARDS. ≈20% of patients rapidly developed COVID‐19‐related ARDS, with mortality rates ranging from 26.0 to 61.5%, making it the primary cause of mortality among these patients.^[^ ^7^, ^8^, ^9^ ^]^

The primary treatments for ALI/ARDS include mechanical ventilation and drug therapy, both of which present significant adverse effects. Mechanical ventilation enhances gas exchange, alleviates respiratory distress, and augments lung compliance. However, it can cause gas accumulation in the alveoli and pulmonary interstitium, resulting in ventilator‐induced lung injury, thereby hampering the disease treatment.^[^ ^10^ ^]^ Anti‐inflammatory drugs for the treatment of ALI, such as antibiotics and glucocorticoids, effectively suppress inflammation and lung injury, but prolonged use may result in severe side effects such as immunosuppression, secondary infections, hyperglycemia, osteoporosis, and even femoral head necrosis. Long‐term antibiotic use can induce drug resistance and gastrointestinal disorders.^[^ ^11^ ^]^ Consequently, identifying safer and more effective therapeutic options for ALI/ARDs is urgently needed.

Extracellular vesicles (EVs) are lipid bilayer‐enclosed vesicles secreted by various cell types. Based on their biological origin and secretory pathways, EVs can be classified into three types: apoptotic bodies (100–5000 nm), originating from cells undergoing apoptosis; microvesicles (MVs) (100–1000 nm), formed through plasma membrane budding and blistering; and exosomes (30–150 nm), resulting from endosomal inward budding and plasma membrane fusion. Due to the challenges in determining the biological origin of EVs, they are often categorized by size: small EVs (sEVs < 200 nm) and medium/large EVs (m/lEVs > 200 nm).^[^ ^12^ ^]^ In this review, the terms “exosome” and “sEVs” are used interchangeably as “EVs”. EVs carry various molecules, including lipids, RNA (messenger RNA (mRNA), microRNA, IncRNA), and proteins, facilitating intercellular communication and material exchange,^[^ ^13^ ^]^ and play pivotal regulatory roles in numerous diseases. Various cell types in lung tissue, including alveolar epithelial cells (ECs), microvascular endothelial cells, neutrophils, and macrophages, can facilitate the onset and progression of ALI/ARDS via EV secretion. EVs loaded with active substances show promise as therapeutic agents for ALI/ARDS.^[^ ^14^ ^]^ Moreover, supportive therapy and regular medication can alleviate symptoms and maintain vital signs, while EVs exert a proactive therapeutic effect by carrying bioactive molecules and directly participating in injury repair and regeneration. Preclinical studies have suggested that EVs from various cell sources can mitigate lung injury through different mechanisms such as suppressing inflammation and repairing the pulmonary air‐blood barrier in animal models.^[^ ^15^ ^]^

However, natural EVs face limitations, including a short in vivo half‐life and rapid accumulation in the liver following intravenous administration, which limits their sustained therapeutic effect on damaged lung tissue.^[^ ^16^ ^]^ Additionally, the heterogeneity of EVs, due to varying cell sources, complicates the regulation of active substance concentrations, hindering optimal therapeutic effects.^[^ ^17^ ^]^ To address these challenges, researchers have developed engineered EVs, which are modified to enhance therapeutic efficiency, enable targeted organ therapy, and extend circulation time in vivo. These EVs are artificially modified to meet research needs. By modifying the surface or interior of natural EVs, their original biological properties can be altered to enhance their clinical potential for the treatment of ALI/ARDS.

In this review, we explore the biological characteristics of EVs, including their origin, membrane composition, and loading contents. We also discuss the therapeutic potential of natural EVs and the application of engineered EVs in ALI/ARDS and have summarized the potential developmental directions of EV‐based therapies for ARDS.

## 2 The Biology of EVs

In this section, we briefly describe the biogenesis and properties of EVs. The biogenesis of EVs is a complex and delicate process involving multiple stages and various regulatory mechanisms. EVs from different sources vary in properties, which lays the foundation for intercellular transport and communication and provides potential for use as therapeutic agents. Under pathological conditions, the number and properties of EVs released by cells differ from those in healthy states, and they play a promoting role in the development of ALI/ARDS. The variability of their properties (membrane composition and luminal contents) also provides a pivot for the engineering technology of EVs.

### 2.1 EV Biogenesis

EVs are classified into exosomes, MVs, and apoptotic bodies based on their origin. Exosomes originate from early endosomes that are formed from the inward budding of the plasma membrane, budding into the endosomal lumen, and forming intraluminal vesicles (ILVs). Subsequently, these ILVs mature into multivesicular bodies (MVBs) with a multivesicular structure.^[^ ^18^, ^19^ ^]^ Some MVBs fuse with the plasma membrane, releasing ILVs into the extracellular space as exosomes, while others are degraded within the cell by lysosomes or autophagosomes.^[^ ^20^ ^]^

Exosome biogenesis involves three primary stages: ILV formation, MVB trafficking, and exosome release. The endosomal sorting complex required for transport (ESCRT) is the primary mechanism involved in this process.^[^ ^21^ ^]^ ESCRT consists of four protein complexes (ESCRT‐0, ESCRT‐I, ESCRT‐II, and ESCRT‐III) that regulate ILV sorting and formation.^[^ ^22^ ^]^ ESCRT‐0, characterized by multiple ubiquitin‐binding sites, is responsible for recruiting, SEQUESTering ubiquitinated cargos, and recruiting ESCRT‐I to the endosome membrane.^[^ ^23^ ^]^

ESCRT‐I recruits ESCRT‐II through the connection of various subunits, primarily mediating membrane curvature. Subsequently, ESCRT‐II completes ILV formation by facilitating vesicle scission.^[^ ^24^ ^]^ Non‐ESCRT‐dependent pathways, including the syndecan‐syntenin‐ALIX pathway,^[^ ^25^ ^]^ lipids,^[^ ^26^, ^27^ ^]^ tetraspanin,^[^ ^28^, ^29^ ^]^ and Rab proteins,^[^ ^30^ ^]^ also contribute to ILV formation. MVB trafficking to the plasma membrane involves interactions with the cytoskeleton, motor proteins, and small GTPases.^[^ ^31^ ^]^ Exosome release is mediated by the soluble N‐ethylmaleimide‐sensitive factor attachment receptor (SNARE) protein complex, where v‐SNARE located on the MVB surface binds to t‐SNARE on the plasma membrane, forming the SNARE complex and promoting MVB fusion with the plasma membrane, ultimately releasing ILVs and resulting in exosome release.^[^ ^32^ ^]^

MV biogenesis occurs in three stages: formation, budding, and secretion. The formation of MVs, akin to the creation of ILVs, can occur through ESCRT‐dependent^[^ ^33^ ^]^ and lipid‐dependent pathways.^[^ ^34^ ^]^ The budding process is triggered by Ca^2+^‐dependent cytoskeletal reorganization, resulting in the formation of MVs in the plasma membrane.^[^ ^35^, ^36^ ^]^ In the final secretion stage, the release of MVs is facilitated by the involvement of Rho family small G proteins, and Rho‐associated kinase (ROCK) participates in the release of MVs.^[^ ^37^ ^]^

The biogenesis of EV is closely related to intracellular transport and metabolic processes. In normal physiological conditions, cells continuously secrete EV to maintain intercellular communication and homeostasis. When disease occurs, the secretion of EV increases significantly.

In the mouse model of ALI induced by intratracheal instillation of LPS, the researchers observed that alveolar macrophages first released EVs in large numbers and peaked. In ventilator‐associated lung injury, EVs released by endothelial cells aggravated lung inflammation and edema and decreased oxygenation index. In ARDS caused by nonbacterial infectious factors (hyperoxia, acid inhalation, influenza A virus), alveolar ECs were the main source of EVs in BALF.^[^ ^38^, ^39^, ^40^ ^]^ Visibly, as an essential intercellular communication medium, EVs originating from different cells can be used not only as biomarkers of ARDS under different inducements but also as an important target for future clinical treatment.

### 2.2 EV Properties

A brief overview of the biophysical properties of EVs is provided below. These properties can be altered using engineering techniques, which are discussed in Section 4.

#### 2.2.1 EV Membrane Composition

Lipids are crucial for maintaining the integrity of EV membranes. Compared with the parent cells, EV membranes exhibit higher cholesterol, sphingomyelin, glycosphingolipids, and phosphatidylserine, but lower levels of phosphatidylcholine.^[^ ^37^ ^]^ Elevated sphingolipid and cholesterol content enhances EV resistance to the harmful effects of detergents and high temperatures. The lipid bilayer of EVs is asymmetrically distributed, with sphingolipids and phosphatidylcholine predominantly occupying the outer leaflet and other lipids primarily concentrated in the inner leaflets.^[^ ^41^ ^]^ Certain lipids (e.g., lipid precursor hexadecylglycerol,^[^ ^42^ ^]^ PI(3,5) P2^[^ ^43^ ^]^) and lipid metabolic enzymes (e.g., neutral sphingomyelinase,^[^ ^25^ ^]^ phospholipase D2,^[^ ^44^ ^]^ diacylglycerol kinase α^[^ ^45^ ^]^) play an important role in the formation and release of EVs.^[^ ^41^, ^46^, ^47^ ^]^

The lipid bilayer of EVs comprises diverse transmembrane and lipid‐binding membrane proteins. Tetraspanins (e.g., CD9, CD63, and CD81) are abundantly expressed in exosomes, contributing to exosome biogenesis and serving as key markers.^[^ ^28^ ^]^ Furthermore, MVs are rich in integrins, selectins, and CD40 ligands. Because EVs originate from the cell plasma membrane, they exhibit heterogeneity, reflecting the specific transmembrane protein receptors^[^ ^48^ ^]^ and adhesion proteins^[^ ^49^ ^]^ of the source cells. These proteins are implicated in essential physiological processes and disease pathogenesis, often serving as important bio‐markers.^[^ ^50^ ^]^

Furthermore, the surfaces of EVs contain a diverse array of glycans and glyco‐binding proteins, including lectins, N‐glycoproteins, O‐glycoproteins, proteoglycans, and glycosphingolipids. These glycoconjugates are crucial for EV transport, internalization, and biogenesis.^[^ ^51^, ^52^ ^]^

#### 2.2.2 EV Lumen

EVs, as crucial mediators of intracellular signaling, harbor a diverse array of proteins and nucleic acid components within their vesicles, which contribute significantly to disease development. Numerous functional proteins have been identified within EVs through techniques such as mass spectrometry and flow cytometry. These proteins, which are integral to vesicle formation and transport, often include ESCRT proteins (such as Alix, TSG101, and VSP40), Syntenin‐1, Rabs, and annexins. These markers are commonly used to identify EVs. Additionally, EVs contain cytoskeletal proteins (e.g., actin and tubulin), molecular chaperones (e.g., HSC70, HSP84), metabolic enzymes (e.g., enolase, GAPDH), and ribosomal proteins.^[^ ^12^ ^]^ The specific proteins found in EVs can vary based on the cellular origin of the vesicles.

Beyond protein components, EVs also carry various forms of nucleic acids, playing a role in the intercellular transfer of genetic material. The RNA encapsulated in EVs is typically short, often less than 200 nucleotides,^[^ ^53^ ^]^ and primarily consists of noncoding RNAs such as microRNAs, tRNA, RNAs, and fragmented mRNA. However, some mRNA up to 1 kb in length have also been found. By delivering these RNA molecules to recipient cells, EVs influence gene expression, making them key players in intercellular communication.

EVs also carry DNA fragments derived from the parent cell's genome, ranging from 100 base pairs (bp) to 2.5 kilobase pairs (kbps) in length. These DNA fragments often represent the entire genome and can reveal mutations present in the parental tumor cells within EVs. The quantity and nature of the nucleic acids in EVs are often influenced by the pathological state of the parent cell, rendering them valuable biomarkers for disease detection.

In fact, during the pathological process of ALI/ARDS, EVs released by alveolar ECs, pulmonary microvascular endothelial cells (PMVECs), alveolar macrophages, and neutrophils contribute to disease progression by carrying specific cargo that disrupts the alveolar‐capillary barrier and recruits and activates immune cells in the lung. Given their roles in these processes, EVs are emerging as valuable tools for diagnosing pathological pulmonary damage and predicting disease progression.^[^ ^13^ ^]^

## 3 Native EVs as Therapeutic for ALI/ARDS

**Figure 1:**
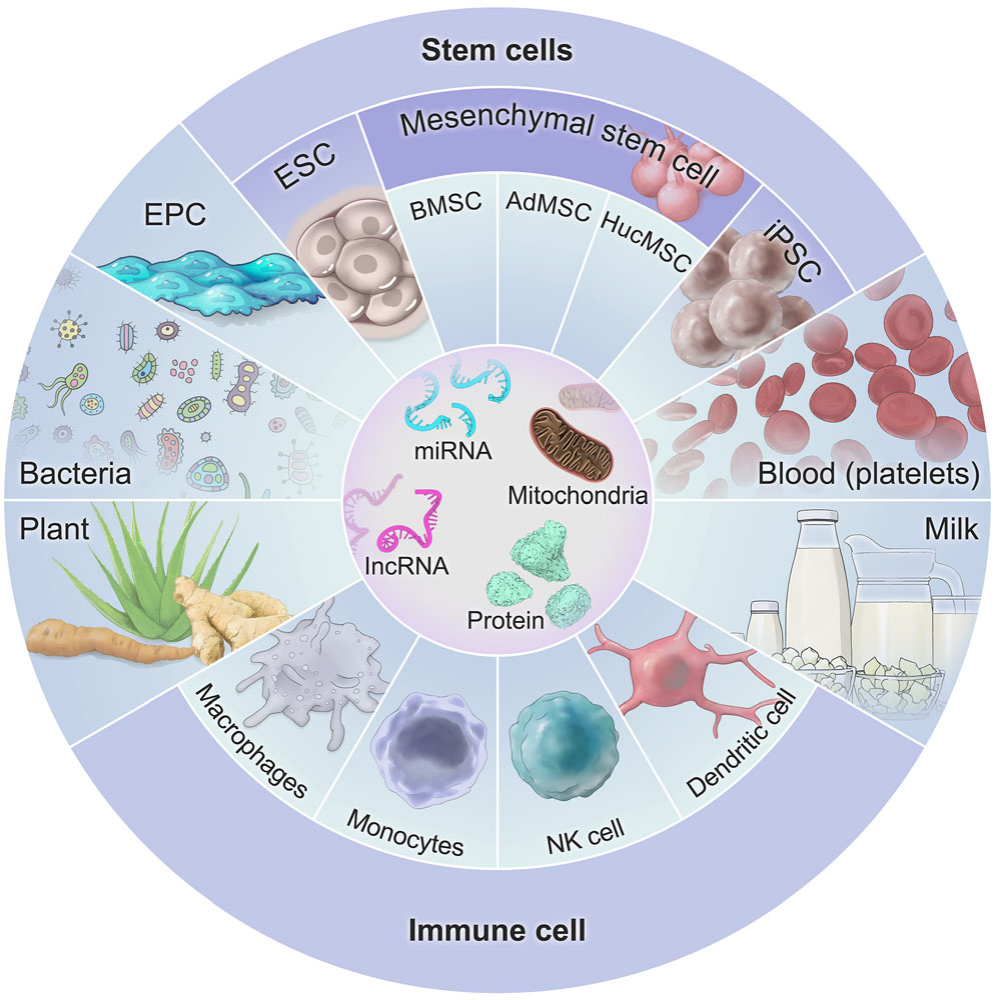
Different sources of EVs.

EVs play a crucial role in intracellular signaling, regulating physiological and pathological processes in lung tissues.^[^ ^54^ ^]^ The sources of EVs are abundant, such as mesenchymal stem cells (MSCs), endothelial progenitor cells (EPCs), macrophages, platelets, plants, and even bacteria^[^ ^55^ ^]^ (**Figure** **1**). Since 2014, several reports have detailed the application of EVs in the treatment of ALI/ARDS. The pathogenesis of ALI/ARDS^[^ ^2^ ^]^ is complex and unfolds in three stages, namely acute exudation, during which immune cells in the lungs are activated by various risk factors, releasing inflammatory mediators that trigger a cascade of responses, ultimately damaging the alveolar epithelium and pulmonary microvasculature; proliferation and repair, where immune cells transition from a proinflammatory to an anti‐inflammatory phenotype, and type II alveolar ECs and lung interstitial fibroblasts proliferate, promoting tissue repair and regeneration; and fibrosis, where excessive proliferation of type II alveolar ECs and fibroblasts leads to pulmonary fibrosis.

Numerous preclinical and clinical studies have reported the safety profile and significant therapeutic efficacy of EVs in treating ALI/ARDS. Compared to traditional cellular therapies, EVs offer several advantages, including small size, low immunogenicity, high stability, regenerative capabilities, and anti‐inflammatory properties. These qualities have made EVs a significant alternative to cellular therapy over the past decade.^[^ ^56^ ^]^ The subsequent discussion will explore the therapeutic potential of EVs derived from natural cells for ALI/ARDS treatment (**Table** **1**).

**Table 1:** Native EVs for the treatment of ALI/ARDS animal models.

Sourcesa)	EV types	Animal model	Administration	Dosing scheme	Mechanism of EVs	References
BMSCs	Exosome	LPS‐induced ALI model (mouse)	I.V.	1 injection of 2 × 10^9^ exosomes in phosphate‐buffered saline (PBS) at 2 h p.d.i	Transfer of miR‐125b‐5p in EVs targeted STAT3 inhibiting macrophage pyroptosis and alleviating sepsis‐associated ALI	[57]
BMSCs	Exosome	LPS‐induced ALI model (mouse)	I.T.	Intratracheal administration of 100 μg exosomes in PBS 24 h before the administration of LPS.	Transfer of miR‐127‐5p in EVs targeted CD64 reducing NETs formation	[58]
BMSCs	Exosome	SM‐induced ALI (mouse)	I.V.	2 injection of 400 μg BMSC‐EVs in PBS at 24 and 72 h p.d.i	Upregulated GPRC5A promoting the expression of Bcl‐2 and junction proteins via regulating the YAP pathway.	[64]
BMSCs	Exosome	LPS‐induced ALI model (mouse)	I.T.	1 injection of 50/100 μg exosomes in PBS at 1 h p.d.i	Inhibited HIF‐1α to down‐regulate the expression of several essential proteins of glycolysis	[89]
BMSCs	Exosome	CLP‐ and LPS‐induced ALI model (mouse)	I.V.	1 injection of 40 μg BMSCs‐exosomes at 4 h pdi	Delivered SAA1 to inhibit CLP‐ or LPS‐induced lung injury and decreased CLP‐ or LPS‐induced endotoxin, TNF‐α, and IL‐6 levels.	[105]
BMSCs	Exosome	Hyperoxia‐induced lung injury model (rat)	I.V.	1 injection of 800 μg BMSCs‐Exos	Transfer of miR‐425 in BMSCs‐Exos targeted PTEN and upregulated the PI3K/AKT axis	[68]
adMSCs	Exosome	LPS‐induced ALI model (mouse)	I.V.	1 injection of 2 μg exosomes in PBS at 4 h p.d.i	Donated mitochondria component to improve macrophage mitochondrial integrity and oxidative phosphorylation level	[107]
adMSCs	Exosome	CLP‐induced sepsis mouse model	I.V.	1 injection of 100 μg exosomes in PBS immediately p.d.i	Transfer of miR‐125b‐5p in EVs regulated Keap1/Nrf2/GPX4 expression alleviating the inflammation induced PMVECs ferroptosis	[59]
adMSCs	sEVs	LPS‐induced ALI model (mouse)	I.V.	1 injection of 20 μg at 30 min p.d.i	Transfer miRNA in EVs promoted autophagy	[173]
hucMSCs	sEVs	SM‐induced ALI (mouse)	I.V.	2 injection of 3 × 10^8^ particle EVs in PBS at 24 and 72 h p.d.i	miR‐146a‐5p delivered by hucMSC‐EVs targeted TRAF6, causing hucMSC‐EVs to exert anti‐inflammatory effects in SM‐induced ALI.	[60]
hucMSCs	sEVs	SM‐induced ALI (mouse)	I.V.	2 injection of 3 × 10^8^ particle EVs in PBS at 24 and 72 h p.d.i	miR‐199a‐5p in HMSCs‐Ex attenuated SM‐associated oxidative stress via regulating CAV1/NRF2 signaling pathway	[65]
hucMSCs	sEVs	LPS‐induced ALI model (mouse)	I.V.	1 injection of 40 μg exosomes in PBS at 6 h p.d.i	sEVs transfer of microRNA‐223‐3p to lung ECs attenuates inflammation in ALI in mice	[61]
hucMSCs	Exosome	LPS‐induced ALI model (mouse)	I.T.	Intratracheal administration of 2 × 10^5^ exosomes in PBS at 4 h p.d.i	miR‐377‐3p released by hucMSCs‐exosomes ameliorated lipopolysaccharide‐induced ALI by targeting RPTOR to induce autophagy in vivo and in vitro.	[73]
hucMSCs	Exosome	LPS‐induced ALI model	–	–	miR‐335‐5p derived from HucMSC‐Exo could alleviate LPS‐induced ALI by regulating the m6A modification of ITGβ4	[179]
MSCs	sEVs	LPS‐induced ALI model (mouse)	I.V.	1 injection of EVs in PBS at 4 h p.d.i	Transferred mitochondrial to restore mitochondrial functions improving alveolar–capillary barrier	[109]
MSCs	Exosome	LPS‐induced ALI model	–	–	lncRNA‐p21‐induced downregulation of miR‐181 might suppress EC apoptosis and alleviate lung tissue injury by upregulating SIRT1 expression	[101]
MSCs	sEVs	LPS‐induced ALI model (mouse)	I.V.	1 injection of 1 × 10^6^ at 4 h p.d.i	Transfer of miR‐181a in EVs downregulated PTEN and subsequently activated pSTAT5 leading to upregulation of SOCS1 in macrophages	[174]
MSCs	sEVs	LPS‐induced ALI model (mouse)	Inhalation	50 μg MSC‐EVs were administered by inhalation after 3 h of LPS induction.	–	[92]
MSCs	sEVs	Influenza virus‐induced ALI model (pig)	I.T.	Intratracheal administration of 80 μg kg^−1^ body weight (12 kg) MSC‐EVs 12 h after influenza virus infection	Reduced virus shedding in the nasal swabs, influenza virus replication in the lungs, and virus‐induced production of proinflammatory cytokines in the lungs of influenza‐infected pigs.	[175]
MSCs	sEVs	Hemorrhagic shock‐induced lung injury model (mouse)	I.V.	1 injection of 30 μg MSC‐EVs in PBS after the shock period	Modulate cytoskeletal signaling and attenuate lung vascular permeability after HS	[176]
MSCs	Exosome	Burn‐induced ALI model (rat)	I.V.	–	HUCMSC‐Exo‐derived miR‐451 improves ALI via the TLR4/NF‐κB pathway	[69]
MSCs	Exosome	Burn‐induced ALI model (rat)	I.V.	–	hUC‐MSCs‐derived exosomal miR‐451 alleviated ALI by modulating macrophage M2 polarization via regulating MIF‐PI3K‐AKT signaling pathway	[180]
MSCs	sEVs	*Escherichia coli*‐induced ALI model (rat)	Inhalation	1 × 10^9^ EVs were administrated by inhalation at 1 h p.d.i	MSC–EVs given IV attenuated LPS‐induced lung injury, and nebulization of MSC–EVs did not affect their capacity to attenuate lung injury caused by *E. coli* pneumonia, as evidenced by reduction in bacterial load and improved lung physiology	[66]
MSCs	sEVs	–	–	–	miR‐34c in MSC‐sEVs can attenuate edematous lung injury via enhancing γ‐ENaC expression, at least partially, through targeting MARCKS and activating the PI3K/AKT signaling pathway subsequently.	[181]
MSCs	sEVs	*Pseudomonas aeruginosa*‐induced lung injury model (mouse)	Inhalation	2 × 10^5^, 6 × 10^5^, 1 × 10^6^, 2 × 10^6^, 6 × 10^6^, or 1 × 10^7^ particles were administered by inhalation at 2 h p.d.i	–	[93]
MSCs	sEVs	LPS‐induced ALI model (mouse)	I.V.	1 injection of 15 μg EVs in PBS	MSC‐EVs delivered at 24 h (as opposed to 0.5, 5, or 10 h) after disease induction resulted in a 2.7–4.4‐fold higher lung uptake of EVs. Biodistribution studies comparing organ‐specific MSC‐EV uptake showed progressive lung accumulation up to 48 h postdelivery	[70]
MSCs	sEVs	LPS‐induced ALI model (mouse)	I.T/I.V.	1 injection of 50 μg at 30 min p.d.i	Transfer of miR‐27a‐3p in EVs targeted NFKB1 promoting M2 macrophage polarization	[177]
iPSC‐Derived MSCs	sEVs	CLP‐induced sepsis mouse model (rat)	I.P.	iMSC‐sEV (2 mg kg^−1^) was intraperitoneally administered 4 h after performing CLP in the rats.	iMSC‐sEV transmitted miR‐125b‐5p into LPS‐treated AMs to target TRAF6	[86]
EPCs	sEVs	CLP‐induced sepsis mouse model (mouse)	I.V.	1 injection of exosomes in PBS at 4 h p.d.i	EPC‐derived EVs transmit TUG1 to attenuate sepsis via macrophage M2 polarization. IncRNA	[102]
EPCs	Exosome	CLP‐induced sepsis mouse model (mouse)	I.T.	Intratracheal administration of 80 μg Exos in PBS at 1 h p.d.i	EC‐derived exosomes overexpressed miR‐125b‐5p to protect from sepsis‐induced ALI by inhibiting TOP2A	[88]
EPCs	Exosome	LPS‐induced ALI model (mouse)	I.T.	Intratracheal administration of 70 μg EPC exosomes in PBS at 4 h p.d.i	The delivery of miRNA‐126 into the injured alveolus.	[90]
EPCs	Exosome	CLP‐induced sepsis mouse model (mouse)	I.V.	1 injection of 40 μg exosomes in PBS at 4 h p.d.i	EPC exosomes prevent microvascular dysfunction and improve sepsis outcomes potentially through the delivery of miR‐126	[178]
EPCs	Exosome	LPS‐induced ALI model (rat)	I.V.	1 injection of 100 μg exosomes in PBS	miR‐126 transferred to target endothelial cells resulted in subsequent downregulation of SPRED1 and promoted RAF/ERK signaling pathways and subsequent improvement in endothelial cell function	[74]
Ginger	ELNs	SARS‐CoV‐2 virus infection (mouse)	I.T.	The exosomes or GNVs (5 × 108 kg^−1^, body weight, *n* = 5 per group) were dispensed into the lung in a single fluid motion.	The role of GELNs in inhibition of the SARS‐CoV‐2‐induced cytopathic effect (CPE) was further demonstrated via GELN aly‐miR396a‐5p and rlcv‐miRrL1‐28‐3p‐mediated inhibition of expression of Nsp12 and spike genes	[76]
R. Radix	ELNs	LPS‐induced ALI model (mouse)	I.G.	–	miR‐7972 derived from fresh R. Radix alleviated LPS‐induced lung inflammation by targeting the GPR161‐mediated Hedgehog pathway, recovering gut microbiota dysbiosis.	[79]
Aloe	ELNs	–	–	–	Reprograming macrophage phenotypes	[78]
Serum	sEVs	LPS‐induced ALI model (rat)	I.V.	1 injection of 70 μg EVs in PBS at 12 h p.d.i	Serum‐derived EV‐loaded miR‐142‐5p downregulated PTEN and activated PI3K/Akt to inhibit ALI in sepsis	[85]
vascular endothelial cells (EnCs) and type II alveolar ECs	Exosome	LPS‐induced ALI model (mouse)	I.T.	The LPS‐induced ALI model: 12.5 μg of CD31 + EXOs; BLM‐induced PF model: 25 μg of CD74 + EXOs	MiR‐223 and miR‐27b‐3p target RGS1	[82]
NK cells	Exosome	*P. aeruginosa*‐induced lung injury model (mouse)	I.V.	–	NK cell may improve PA‐induced lung injury through promoting M1 lung macrophage polarization by secreting exosome	[87]
Alveolar progenitor type II cell (ATIIC)	Exosome	Bleomycin‐induced lung injury model	–	–	PTEN as a direct target of miR‐371b‐5p	[83]
Serum	sEVs	LPS‐induced ALI model (mouse)	Inhalation	After 2 h, mice were given sEVs via nebulizer as indicated. 250 μg DiR‐labeled sEVs were delivered to the mice through inhalation using FlexiVent FX 2 system	–	[94]
menstrual blood‐derived stem cells	sEVs	LPS‐induced ALI model (mouse)	I.T.	Intratracheal administration of 1 × 10^6^ MenSC‐EVs after 4 h LPS induction	MiR‐671‐5p directly targets the kinase AAK1 for post‐transcriptional degradation. AAK1 is found to positively regulate the activation of nuclear factor kB (NF‐kB) signaling by controlling the stability of the inhibitory protein IkBa	[62]
Adipose	Exosome	Ventilator‐induced lung injury model (mouse)	I.V.	0, 25, 50, and 100 μg mL^−1^ in a total volume of 200 μL of PBS 1 h before MV to determine the optimal exosome intervention concentration	Inhibiting the TRPV4/ Ca2 signaling pathway	[84]

a) I.T.: intratracheal injection; p.d.i: postdisease induction; CLP: cecum ligation and puncture; SM: sulfur mustard.

### 3.1 EV Source

EVs act as paracrine agents, carrying various donor cell components and mirroring the therapeutic effects of these cells. Several studies have demonstrated that EVs from natural sources play a pivotal role in ameliorating ALI/ARDS.

Early research predominantly focused on EVs derived from MSCs and their therapeutic potential in ALI/ARDS. These MSC‐EVs carry various effector molecules that enable them to mimic the biological effects of their parent MSCs.^[^ ^56^ ^]^ MSC‐EVs sourced from bone marrow,^[^ ^57^, ^58^ ^]^ adipose tissue,^[^ ^59^ ^]^ human umbilical cord,^[^ ^60^, ^61^ ^]^ and menstrual blood^[^ ^62^ ^]^ have good antibacterial activity, antiviral activities,^[^ ^63^ ^]^ barrier repair, immune regulation, and autophagy modulation, demonstrating a range of beneficial effects in ALI/ARDS. In animal models, MSC‐EVs have demonstrated significant therapeutic effects in ALI/ARDS induced by a variety of triggers, including mustard gas,^[^ ^64^, ^65^ ^]^ sepsis,^[^ ^59^ ^]^ endotoxins,^[^ ^14^ ^]^ bacterial^[^ ^66^, ^67^ ^]^ and viral infections,^[^ ^66^ ^]^ hyperoxia,^[^ ^68^ ^]^ and burns.^[^ ^69^ ^]^ Furthermore, in ALI animal models, MSC‐EVs can target lung tissue. Following systemic administration, MSC‐EVs accumulate in lung tissue, peaking at around 48 h postdelivery, with a decline by 72 h. Compared with EVs derived from human embryonic kidney 293 cells (HEK293T) cells, the accumulation of MSC‐EVs in the lung tissue was significantly higher.^[^ ^70^ ^]^

EPCs, the precursors of endothelial cells, exhibit stem cell characteristics and play a pivotal role in endothelial cell regeneration and vascular repair.^[^ ^71^, ^72^ ^]^ EPC‐derived EVs (EPC‐EVs) released via the paracrine pathway mimic these regenerative effects on endothelial barrier repair. In both animal and cell models of ALI/ARDS, EPC‐EVs promote the expression of endothelial nitric oxide synthase (eNOS), vascular endothelial growth factor (VEGF), and vascular endothelial growth factor receptor 2 (VEGFR‐2). This is primarily achieved through the transfer of various factors (microRNA (miRNA)‐126,^[^ ^60^ ^]^ miR‐126‐3p, miR‐126‐5p,^[^ ^61^, ^65^ ^]^ long noncoding RNA (lncRNA)‐TUG1^[^ ^73^ ^]^). EPC‐EVs also promote angiogenesis, inhibit endothelial cell apoptosis, and restore endothelial barrier integrity.^[^ ^74^, ^75^ ^]^

Plant‐derived extracellular vesicle‐like nanoparticles (ELNs) offer unique therapeutic potential in the treatment of ALI/ARDS. These naturally occurring nanoscale vesicles, extracted from plants, possess similar physical properties to EVs derived from animal cells and contain various unique bioactive components.^[^ ^76^, ^77^ ^]^ ELNs derived from plants, including Aloe vera,^[^ ^78^ ^]^ fresh Rehmannia glutinosa,^[^ ^79^ ^]^ and ginger,^[^ ^76^ ^]^ participate in inflammation regulation and antiviral activities in ALI due to their plant‐derived miRNAs, effectively inhibiting disease progression.

Beyond the aforementioned EVs, EVs derived from red blood cells,^[^ ^80^ ^]^ trophoblasts,^[^ ^81^ ^]^ endothelial cells,^[^ ^82^ ^]^ ECs,^[^ ^83^ ^]^ adipose tissue,^[^ ^84^ ^]^ serum,^[^ ^85^ ^]^ induced pluripotent stem cells (iPSC),^[^ ^86^ ^]^ and natural killer cells^[^ ^87^ ^]^ have shown efficacy in the treatment and prevention of ALI/ARDS. These EVs work by interacting with specific cellular receptors or delivering miRNAs to regulate signaling pathways.

### 3.2 Administration Route

**Figure 2:**
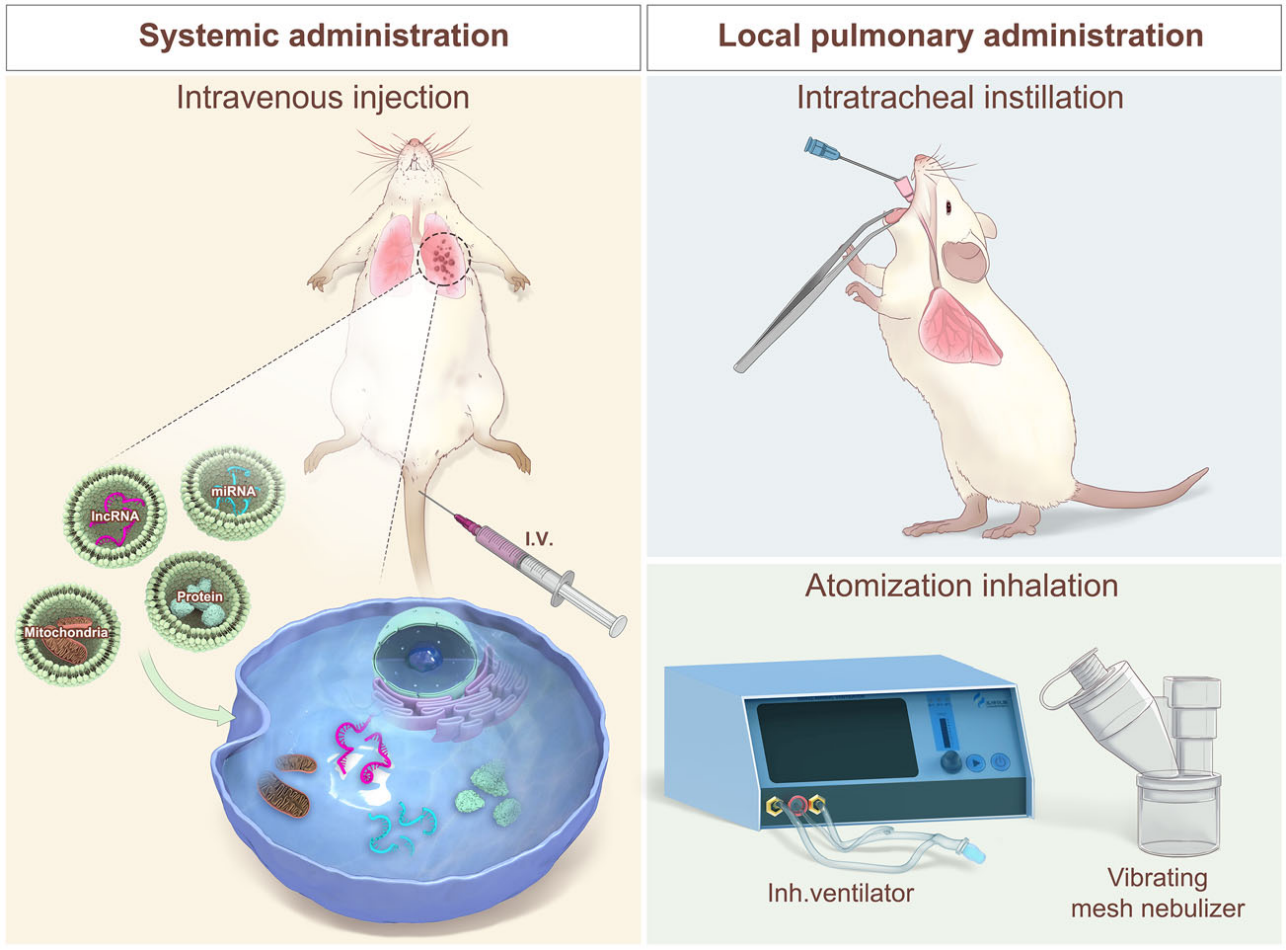
EV administration route in treatment of ALI/ARDS.

The administration route significantly influences the distribution of EVs in vivo. In ALI/ARDS animal models, EVs are typically administered via intravenous injection (I.V.),^[^ ^58^, ^59^, ^70^, ^88^ ^]^ tracheal instillation,^[^ ^57^, ^62^, ^73^, ^89^, ^90^, ^91^ ^]^ or inhalation^[^ ^66^, ^92^, ^93^, ^94^, ^95^, ^96^ ^]^ (**Figure** **2**). However, only a few studies have examined the accumulation of EVs in the lung tissues following different administration routes.^[^ ^92^ ^]^

I.V. is the most prevalent administration route in ALI/ARDS animal models, offering advantages such as precise dosage, simplicity of operation, rapid onset, and high bioavailability. The lung's extensive vascular network, coupled with the compromised air‐blood barrier during ALI, facilitates the passive targeting of EVs to damaged lung tissue. Nonetheless, in vivo distribution studies revealed that natural EVs do not exhibit substantial targeting of the lung tissue. Lázaro‐Ibáñez E^[^ ^16^ ^]^ found that EVs derived from HEK293T cells primarily accumulated in the liver and spleen, with only weak signals detected in the lung tissue 24 h postinjection. Hemodynamic analysis revealed that HEK293T‐EVs were rapidly eliminated from the body, with a half‐life of less than 10 min. However, the in vivo distribution of EVs varies depending on the disease model and the cell source. For example, in LPS‐induced ALI models, MSC‐EVs were found to target lung tissue more effectively. In an LPS‐induced ALI model, compared with I.V. at 0.5, 5, and 10 h after LPS induction, administration at 24 h more effectively promoted the absorption of EVs by the lung tissue. Biodistribution experiments revealed that MSC‐EVs accumulated in lung tissue for 48 h postinjection, gradually decreasing after 72 h with less accumulation in the spleen and liver compared to HEK293T‐EVs.^[^ ^70^ ^]^

Local pulmonary administration, such as intratracheal instillation and nebulization, offers the advantages of rapid absorption, no first‐pass effect, reduced distribution in the liver and spleen, and direct effects on damaged lung tissue over systemic administration. Intratracheal instillation involves injecting EVs directly into the trachea, allowing entry into the lung tissue. Compared with nebulization, intratracheal instillation requires simpler equipment and allows for precise dosage control. In animal models of ALI, intratracheal instillation of EVs exerted immune regulation, autophagy induction, barrier protection, and virus clearance effects. However, intratracheal instillation is invasive and unevenly distributed and requires anesthesia, thereby limiting its clinical usage.^[^ ^97^ ^]^ In recent years, nebulization has gradually become the preferred pulmonary administration route. EVs are dispersed into aerosol particles of 1–5 μm and efficiently delivered to lung tissue non‐invasively. Compared with I.V., inhalation delivery can reach the lung tissue directly, facilitating lung injury repair.^[^ ^92^ ^]^ The vibrating mesh nebulizer is commonly used for EV nebulization, consistently producing aerosol particles below 5 μm, ensuring that EVs reach the bronchioles and damaged lung tissues. In vivo biodistribution experiments on ALI mice nebulized with MSC‐EVs revealed that MSC‐EVs were primarily distributed in the lung tissue, peaking at 24 h postadministration and remaining detectable in the lungs 28 days post‐administration.^[^ ^93^ ^]^ Nebulized MSC‐EVs also effectively reduced inflammation, decreased bacterial load in ALI mice, and improved lung function.^[^ ^68^ ^]^

Moreover, advances in aerosolizable dry powders of EVs prepared by thin‐film freeze‐drying technology have shown promise. These powders retain the original structure and morphology of EVs upon reconstitution, with an optimal mass median aerodynamic diameter (MMAD) of ≈1.2 μm for lung delivery.^[^ ^98^ ^]^ Clinical trials revealed that nebulized MSC‐EVs have good tolerability and short‐term safety^[^ ^93^ ^]^ and do not induce acute or secondary allergic reactions; however, they enhanced the absorption of pulmonary lesions and shortened the hospitalization period in patients with mild COVID‐19 pneumonia.^[^ ^96^ ^]^

### 3.3 EV Dosage

In addition to administration routes and cell sources, EV dosage is a critical factor influencing therapeutic efficacy. To better understand EV dosage and administration strategies in ALI/ARDS, we reviewed 33 preclinical studies on EV‐based treatments of ALI/ARDS (Table 1). This study explored varying EV dosages across different administration routes and animal models to establish an effective dosage range.

**Figure 3:**
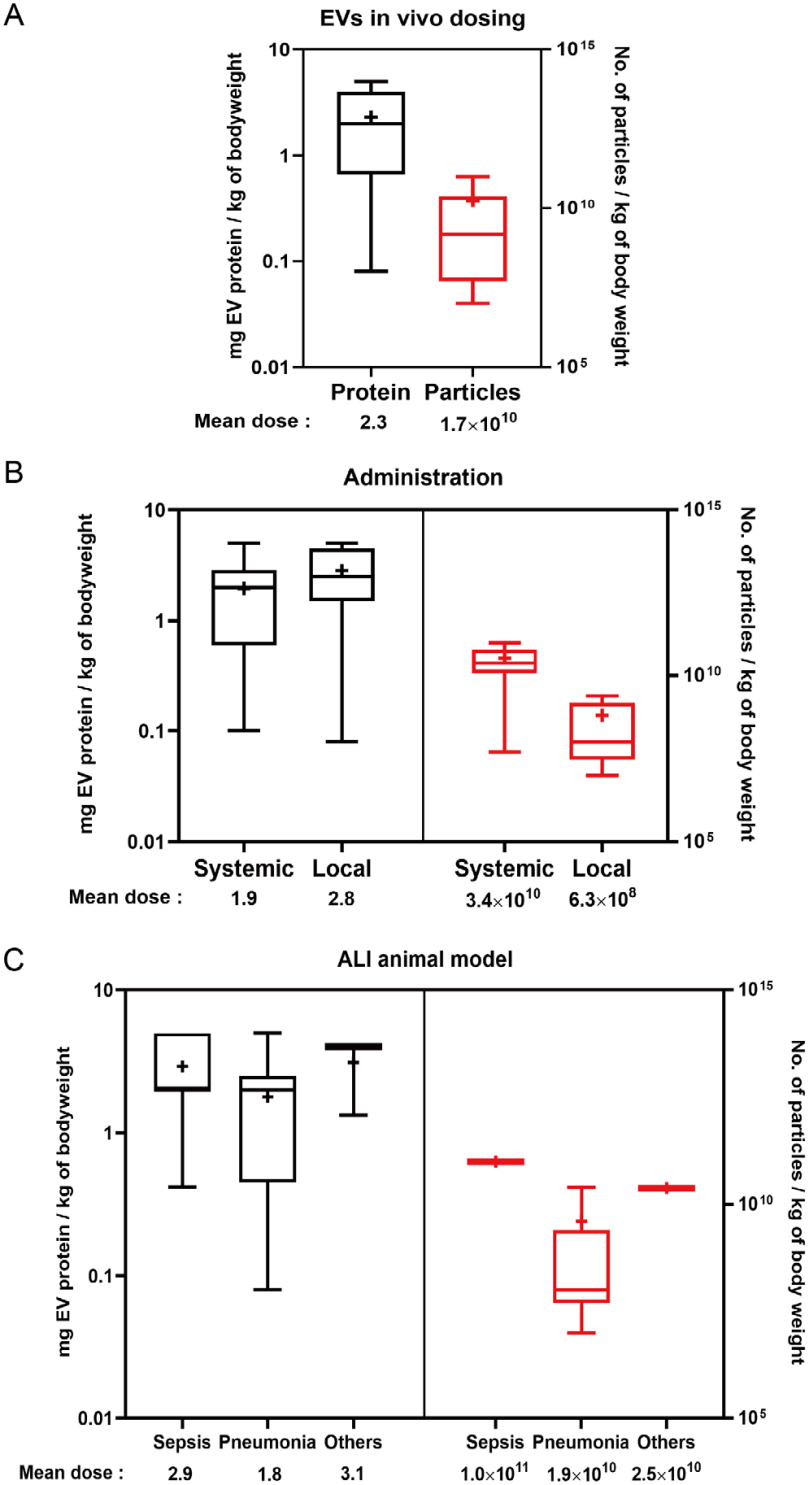
A) Graphical representation of identified EV doses based on protein or particle quantification across 33 different preclinical studies.^[^ ^57^, ^58^, ^59^, ^60^, ^61^, ^62^, ^64^, ^65^, ^66^, ^68^, ^70^, ^73^, ^74^, ^82^, ^84^, ^85^, ^86^, ^88^, ^89^, ^90^, ^92^, ^93^, ^94^, ^102^, ^105^, ^107^, ^109^, ^173^, ^174^, ^175^, ^176^, ^177^, ^178^ ^]^ B) Segregation of EV doses used in different studies based on the route of administration. C) Distribution of EV dosing used in preclinical studies based on the different animal models.

Among the 33 studies mentioned with dosing schemes in Table 1 and 23 quantified EV dosages were based on total protein content, ranging from 0.1 to 5 mg kg^−1^. The remaining 10 studies quantified EV dosage based on the number of particles measured by nanoparticle tracking analysis (NTA), ranging from 1 × 10^7^ to ×10^11^ particles kg^−1^. Compared with particle numbers, studies utilizing protein content quantification showed greater inconsistencies and data dispersion (**Figure** **3**A).

Notably, the protein content of EV samples may vary due to factors such as the source, isolation, and purification methods.^[^ ^99^, ^100^ ^]^ Current clinical studies primarily use particle numbers as a quantitative criterion; therefore, future studies should use particle number quantification to improve consistency.

Different administration routes influence EV dosage requirements. Studies segregated by administration route showed that systemic administration methods, such as intravenous or intraperitoneal injections (I.P.), generally require higher dosages compared to local pulmonary administration routes, such as intratracheal or inhalation (Figure 3B). Additionally, different animal models of ALI and ARDS affect the dosage of EVs. A comparison of the doses of EVs in different ALI/ARDS models showed that higher doses of EVs were usually required in sepsis‐induced ALI models (Figure 3C).

Determining the optimal dosage is a prerequisite for the efficacy of EVs. In a dose‐response study, ALI mice were administered different doses of MSC‐EVs. Within the dose range of 2 × 10^5^–2 × 10^6^ particles, higher doses correlated with improved survival. However, doses exceeding 2 × 10^6^ particles showed a negative correlation with survival rate, indicating that appropriate dosing is important for maximizing the therapeutic effects of EVs.^[^ ^93^ ^]^ Preclinical studies are a prerequisite for clinical research. Thus, improving the evaluation of EV dosages and administration regimens is crucial to facilitate their clinical translation.

### 3.4 Mechanism of Action

EVs mediate cellular signaling in ALI/ARDS by delivering noncoding RNAs, proteins, and mitochondrial components (Table 1).

#### 3.4.1 Noncoding RNAs

Natural EVs mainly convey a variety of noncoding RNAs, such as miRNAs, lncRNAs, and mRNA fragments. Accumulating evidence has demonstrated that noncoding RNAs have participated in various biological processes.

Current studies exploring the role that noncoding RNAs derived from EVs play in the ALI/ARDS area are largely focused on miRNAs. miRNAs are small, endogenous noncoding RNAs, typically 22–26 nucleotides in length that function primarily as post‐transcriptional regulators of gene expression. Numerous studies have reported that miRNAs encapsulated within EVs play pivotal roles in anti‐inflammatory, antioxidative, antiapoptotic processes, and barrier repair within the ALI/ARDS model. For instance, miR‐125b‐5p present in adipose‐derived mesenchymal stem cell (AdMSC)‐EVs could alleviate LPS‐induced PMVEC ferroptosis in sepsis‐induced ALI by regulating the Keap1/Nrf2/GPX4 expression axis.^[^ ^59^ ^]^ miR‐146a‐5p, delivered by hucMSC‐EVs, targets TRAF6, enabling hucMSC‐EVs to exert anti‐inflammatory effects in SM‐induced ALI.^[^ ^60^ ^]^ miR‐223‐3p has been identified as a crucial mediator in the regulatory effects induced by MSC‐EVs through the inhibition of polymerase‐1 in lung ECs.^[^ ^61^ ^]^ In addition, miR‐199a‐5p in HMSCs‐Ex mitigates SM‐associated oxidative stress by modulating the CAV1/NRF2 signaling pathway.^[^ ^65^ ^]^ Bone marrow mesenchymal stem cell (BMSC)‐EVs containing miR‐127‐5p can downregulate CD64 to reduce tissue damage and inhibit the release of inflammatory factors and neutrophil extracellular trap (NET) formation.^[^ ^58^ ^]^ miRNA‐125b‐5p derived from BMSCs‐EVs downregulates STAT3, thus inhibiting macrophage pyroptosis and alleviating sepsis‐associated ALI.^[^ ^57^ ^]^ miR‐377‐3p released by human umbilical cord mesenchymal stem cells (hucMSCs)‐exosomes ameliorated lipopolysaccharide (LPS)‐induced ALI by targeting RPTOR to induce autophagy in vivo and in vitro.^[^ ^73^ ^]^

EVs also transport lncRNAs, which act as competing endogenous RNAs. These lncRNAs indirectly upregulate target genes by binding to miRNAs and diminishing their activities. Sui et al.^[^ ^101^ ^]^ demonstrated that MSC‐EVs could clear miR‐181 in vivo by delivering IncRNA p21, promoting SIRT1 expression and protecting the epithelial barrier. Ma et al.^[^ ^102^ ^]^ discovered that in a mouse model of sepsis, EPC‐EVs competitively bind to IncRNA TUG1 by transporting microRNA‐9‐5p, upregulating SIRT1 expression to promote M2 macrophage polarization, reducing lung tissue injury.

Furthermore, MVs, which are an important component of EVs, exhibit therapeutic potential by transporting mRNA. Tang et al.^[^ ^91^, ^103^ ^]^ reported that MSC‐MVs improve lung inflammation by delivering keratinocyte growth factor (KGF) mRNA and angiopoietin‐1 (Ang‐1) mRNA, restoring the integrity of the air‐blood barrier.

#### 3.4.2 Proteins

The proteins present in EVs constitute key components for their functionality. Hepatocyte growth factor (HGF), which is a crucial factor associated with endothelial permeability, can display barrier‐protective effects on endothelial cells in ALI/ARDS. Hualing Wang et al.^[^ ^104^ ^]^ demonstrated that HGF is a key factor within MSC‐MVs for protecting the endothelial barrier. In the LPS‐induced endothelial cell model, MSC‐MVs could decrease transcellular permeabilities, and the effect was significantly inhibited after HGF gene knockdown in MSC‐MVs. Moreover, HGF within MVs can also act on the endothelial intercellular junction proteins, reduce the apoptosis of endothelial cells, induce the proliferation of endothelial cells, and protect and stabilize the endothelial barrier function. Serum amyloid A1 (SAA1) is an acute‐phase protein, which can regulate inflammation and immunity. Zhou Lv et al.^[^ ^105^ ^]^ showed that BMSCs‐Exo can inhibit sepsis‐induced lung injury through SAA1.

Apoptotic bodies, which are another subset of EVs, can also exert an effect through surface proteins. Apoptotic bodies can initiate immunoregulation to prevent and suppress inflammation and autoimmunity. Jiang et al.^[^ ^106^ ^]^ demonstrated that apoptotic bodies released by UC‐MSCs convert the macrophages from a proinflammatory to an anti‐inflammatory state, thereby ameliorating the disease. They identify the expression of programmed cell death 1 ligand 1 (PDL1) on the membrane of UC‐MSC‐derived ABs, which interacts with programmed cell death protein 1 (PD1) on host macrophages. Such an interaction leads to the reprogramming of macrophage metabolism, causing a shift from glycolysis to mitochondrial oxidative phosphorylation via the Erk‐dependent pathway in ALI.

#### 3.4.3 Mitochondrial Translocation and Repair of Mitochondrial Dysfunction

Mitochondrial translocation and repair of mitochondrial dysfunction represent another mechanism through which EVs exert an effect on ALI/ARDS. During the progression of ALI/ARDS, reactive oxygen species generated by mitochondria induce defects in DNA transcription, thereby resulting in mitochondrial dysfunction. Furthermore, damaged mitochondrial DNA perpetuates adverse reactions within the lungs.

The group showed that EVs derived from AdMSCs transfer mitochondrial components (mtDNA and mitochondrial proteins‐TOM20, NDUFV2) to alveolar macrophages in a dose‐dependent manner. After restoring the integrity of mitochondria, the level of mtDNA, mitochondrial membrane potential, OXPHOS activity, and ATP generation were elevated, and mROS stress was decreased in macrophages. Macrophages shift from a proinflammatory phenotype to an anti‐inflammatory phenotype, thereby leading to the restoration of metabolic and immune homeostasis of airway macrophages and alleviating lung inflammatory pathology.^[^ ^107^ ^]^ Furthermore, EVs originated from BMSCs can deliver mitochondria to alveolar macrophages. This transfer process leads to an enhancement of the oxidative phosphorylation and phagocytic abilities of these macrophages. Moreover, it results in a decrease in the secretion of TNF‐α, thus alleviating inflammation and lung injury in ARDS.^[^ ^108^ ^]^

EVs can also modulate alveolar‐capillary barrier integrity through mitochondrial transfer in the ARDS animal model. Dutra Silva et al.^[^ ^109^ ^]^ reported that in ARDS, EVs derived from normal MSCs restored the integrity of the air‐blood barrier, downregulated the level of ROS, and restored the normal levels of oxidative phosphorylation by transferring healthy mitochondria to damaged alveolar epithelial and endothelial cells, repairing mitochondrial function.

Collectively, these findings provide important evidence for EV‐mediated mitochondrial transfer as a mechanism for the treatment of ALI/ARDS.

### 3.5 Clinical Research

Numerous clinical trials have explored the therapeutic potential of EVs in treating ALI/ARDS, focusing on the safety and efficacy of MSC‐EVs^[^ ^56^ ^]^ (**Table** **2**). One clinical trial evaluated the safety and tolerability of clinical‐grade HAMSC‐EVs in 24 healthy volunteers who inhaled 2 × 10^8^–1.6 × 10^9^ particles. The trial demonstrated good tolerability and short‐term safety, with no severe adverse events or allergic reactions reported within 7 days postadministration (NCT04313647).^[^ ^93^ ^]^ Additionally, the administration of MSC‐EVs to patients with ARDS has shown a high degree of safety. In several clinical studies investigating the use of EVs to treat patients with COVID‐19‐related ARDS, no significant adverse reactions or related adverse events were observed during treatment with varying doses of MSC‐EVs (NCT04491240, NCT04493242, and IRCT20200217046526N2).

**Table 2:** Clinical trials of the therapeutic effects of EVs on ALI/ARDS patients.

Trial identification	Official title	EV source	Disease	Drug delivery protocol	Enrollment	Phase	Status	References
NCT04602104	A Multiple, Randomized, Double‐blinded, Controlled Clinical Study of Allogeneic Human Mesenchymal Stem Cell Exosomes (hMSC‐Exos) Nebulized Inhalation in the Treatment of Acute Respiratory Distress Syndrome	hMSCs‐Exo	ARDS	Phase 1: basic treatment and 7 times aerosol inhalation of hMSC‐Exos at day 1, day 2, day 3, day 4, day 5, day 6, day 7 hMSC‐Exos low dose (2.0 × 10^8^ particles); medium dose (8.0 × 10^8^); high dose (16.0 × 10^8^) Phase 2: Basic treatment and 7 times aerosol inhalation of hMSC‐Exos at day 1, day 2, day 3, day 4, day 5, day 6, day 7 Dosage 1:A quarter of MTD/day; Dosage 2: MTD/day Placebo comparator: Basic treatment and 7 times aerosol inhalation of normal saline (at day 1, day 2, day 3, day 4, day 5, day 6, day 7)	18	1,2	Completed	–
NCT05354141	Bone Marrow Mesenchymal Stem Cell Derived Extracellular Vesicles for Hospitalized Patients With Moderate‐to‐Severe ARDS: A Phase III Clinical Trial	BMSCs EVs	ARDS	Placebo comparator: Intravenous administration of normal saline 100 mL Experimental dose: Intravenous administration of normal saline 85 mL and ExoFlo 15 mL	970	3	Recruiting	–
NCT05387278	Safety and Effectiveness of Placental Derived Exosomes and Umbilical Cord Mesenchymal Stem Cells in Moderate to Severe Acute Respiratory Distress Syndrome (ARDS) Associated With the Novel Corona Virus Infection (COVID‐19)	EV‐Pure and WJ‐Pure	COVID‐19 ARDS	Experimental: The treatment consists of administration of WJ‐Pure and EV‐Pure plus standard care Placebo comparator: Cryopreservation media plus standard care Intravenous infusion	20	1	Suspended	–
NCT04798716	Mesenchymal Stem Cell Exosomes for the Treatment of COVID‐19 Positive Patients With Acute Respiratory Distress Syndrome and/or Novel Coronavirus Pneumonia	MSCs‐Exo	Novel Coronavirus Pneumonia ARDS	Experimental: Escalating Dose First Cohort (intravenously every other day on an escalating dose: 2 × 10^9^, 4 × 10^9^, 8 × 10^9^ mL^−1^. Experimental: Escalating Dose Second Cohort (intravenously every other day on an escalating dose: 8 × 10^9^, 4 × 10^9^, 8 × 10^9^ mL Experimental: Escalating Dose Third Cohort (intravenously every other day on an escalating dose: 8 × 10^9^, 8 × 10^9^, 8 × 10^9^ mL	55	1,2	Unknown status	–
NCT04493242	Bone Marrow Mesenchymal Stem Cell Derived Extracellular Vesicles Infusion Treatment for COVID‐19 Associated Acute Respiratory Distress Syndrome (ARDS): A Phase II Clinical TrialTreatment for COVID19 Associated Acute Respiratory Distress Syndrome (ARDS): A Phase II Clinical Trial	BMSCs‐EVs	COVID‐19 ARDS	Placebo comparator: Intravenous administration of normal saline 100 mL Experimental dose 1: Intravenous administration of normal saline 90 mL and ExoFlo 10 mL Experimental dose 2: Intravenous administration of normal saline 85 mL and ExoFlo 15 mL	102	2	Completed	[112]
NCT04747574	A Phase I Feasibility Study to Evaluate the Safety of CD24 Exosomes in Patients With Moderate/Severe COVID‐19 Infection	Exosomes overexpressing CD24	COVID‐19	Aerosolized in normal saline for inhalation via a standard hospital‐grade inhalation device, QD for 5 days, four doses (1 × 10^8^/5 × 10^8^/1 × 10^9^/1 × 10^10^ exosome particles per 2 mL saline)	35	1	Unknown	–
IRCT20200217046526N2	Allogenic mesenchymal stromal cells and their extracellular vesicles in COVID‐19 induced ARDS: a randomized controlled trial	MSCs EVs	COVID‐19 ARDS	First dose: intravenous infusion of allogenic MSCs at a dose of 100 × 10^6^ ± 10% Second dose: nebulized inhalation of MSC‐EVs (isolated from the 200 × 10^6^ ± 10% cells) 48 h after the first injection	43	2	Completed	–
NCT04969172	A Phase II Randomized, Double‐blind, Placebo‐controlled Study to Evaluate the Safety and Efficacy of Exosomes Overexpressing CD24 to Prevent Clinical Deterioration in Patients With Moderate or Severe COVID‐19 Infection	Exosomes overexpressing CD24	COVID‐19 Disease	The exosomes will be diluted in 4 mL normal saline for inhalation, administered once daily (QD) for 5 days. Placebo (saline) will be prepared for inhalation and administered in the same manner as the exosomes	155	2	Unknown status	–
NCT04276987	A Pilot Clinical Study on Aerosol Inhalation of the Exosomes Derived From Allogenic Adipose Mesenchymal Stem Cells in the Treatment of Severe Patients With Novel Coronavirus Pneumonia	MSCs‐Exo	Coronavirus Pneumonia	Experimental: MSCs‐derived Exosomes Treatment Group. 5 times aerosol inhalation of MSC‐derived exosomes (2.0 × 10^8^ nano vesicles/3 mL at day 1, day 2, day 3, day 4, day 5).	24	1	Completed	[110]
NCT06002841	Extracellular Vesicles From Mesenchymal Cells in the Treatment of Acute Respiratory Failure Associated With SARS‐CoV‐2 and Other Etiologies: a Clinical Trial, Randomized, Double‐blind.	MSCs EVs	ARDS	Experimental: Received two intravenous administration of 25 mL Plasma‐Lyte A solution containing MSCs‐EVs, with a 48 h intervalPlacebo comparator: Intravenous administration of normal saline.	15	1,2	Not yet recruiting	–
NCT05947747	A Phase IIb, Randomized, Double‐blinded, Placebo‐controlled Study to Evaluate the Safety and Efficacy of Exosomes Overexpressing CD24 to Prevent Clinical Deterioration in Patients With Mild‐Moderate ARDS	Exosomes overexpressing CD24	ARDS	Experimental: The exosomes were diluted in 1.5 mL normal saline for inhalation, administered twice a day for 5 days Placebo comparator: Patients received a 5‐day treatment in a clean sterile saline solution, administered twice a day for 5 days	90	2	Recruiting	–
NCT04384445	A Phase I/II Randomized, Double Blinded, Placebo Trial to Evaluate the Safety and Potential Efficacy of Intravenous Infusion of Zofin for the Treatment of Moderate to SARS Related to COVID‐19 Infection vs Placebo	human amniotic fluid	COVID‐19	Experimental: Zofin plus standard care. Zofin will be administered intravenously with 1 mL, containing 2‐5 × 10^11^ particles mL^−1^ in addition to the standard care. The Zofin dose will be diluted in 100 mL of sterile saline at subject's bedside. Placebo comparator: Placebo plus standard care	20	1,2	Completed	[182]

Aerosol inhalation is a commonly used drug delivery method in clinical trials. In a study on patients with severe COVID‐19, seven participants received daily treatment with 2.0 × 10^8^ particles of haMSCs‐Exos for 5 days. The results indicated that all participants exhibited good tolerance to aerosol inhalation, with no adverse events reported, and participants showed varying degrees of improvement in the pulmonary lesions (NCT04276987).^[^ ^110^ ^]^ Another clinical trial involved patients with COVID‐19 who underwent twice‐daily aerosol inhalation of 3.83 × 10^9^–3.50 × 10^8^ MSC‐EVs. This treatment did not induce allergic reactions. After aerosol inhalation of MSC‐EVs, the CRP level in patients was reduced, which promoted the absorption of pulmonary lesions and shortened the hospital stay for patients with mild symptoms (ChiCTR2000030261).^[^ ^96^ ^]^

Intravenous infusion is another crucial route for EV administration in clinical settings. In a clinical study treating severe COVID‐19‐related ARDS with bone marrow‐derived mesenchymal stem cell exosomes (ExoFlo) after intravenous infusion, patients showed significant improvements in clinical status and oxygenation, with a 192% increase in PaO_2_/FiO_2_. Laboratory results showed reduced neutrophils, increased CD3^+^, CD4^+^, and CD8^+^ lymphocyte counts, and decreased acute‐phase reactants such as CRP, ferritin, and d‐dimer.^[^ ^111^ ^]^ In another study, patients with moderate‐to‐severe ARDS received intravenous infusions of placebo, 10 mL of ExoFlo (0.9 × 10^12^), or 15 mL of ExoFlo (1.2 × 10^12^) on days 1 and 4. Both doses of ExoFlo were safe. No treatment‐related adverse events were reported. Compared with placebo and ExoFlo‐10 mL, ExoFlo‐15 mL was more effective, which could notably reduce mortality, shorten the time to hospital discharge, and improve ventilation‐free days (NCT04493242).^[^ ^112^ ^]^

The results from published clinical trials suggest that EVs are a promising therapeutic candidate for patients with ALI/ARDS, though they still face limitations and challenges. The origins of EVs are diverse and heterogeneous, and the active ingredients that they contain are also different. Hence, the findings from the aforementioned clinical study cannot be generalized as class effects. Current clinical studies focus on the safety and preliminary efficacy of EVs. Therefore, large‐scale, multicenter, and long‐term follow‐up clinical trials are necessary to assess the potential benefits of EVs for ALI/ARDS in the future.

## 4 Application of Engineered EVs in the Treatment of ALI/ARDS

**Figure 4:**
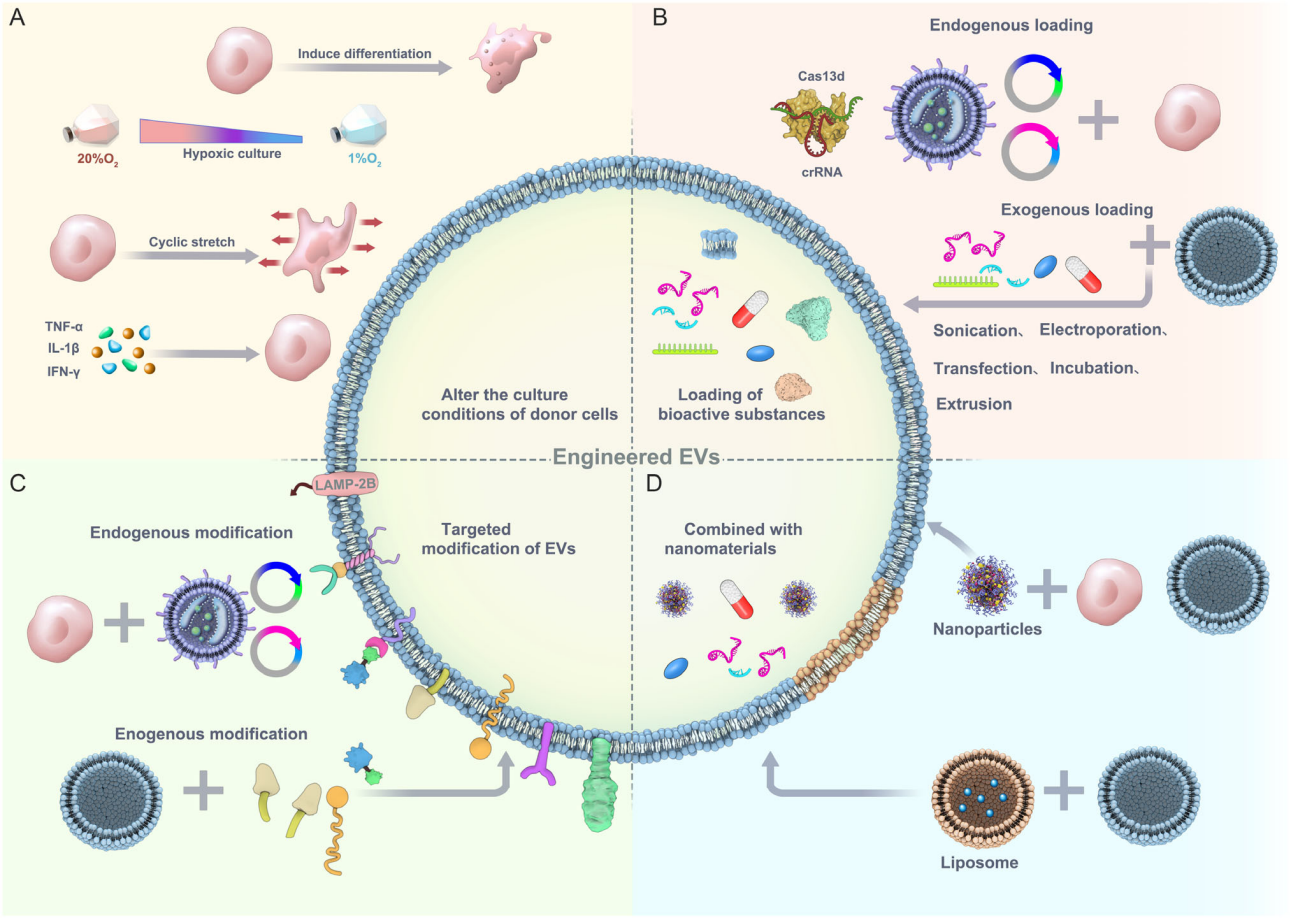
Strategies for engineering therapeutic EVs. A) Alter the culture conditions of donor cells. The biological activity of EVs secreting donor cells can be modulated by adjusting the culture conditions, such as hypoxia culture, cyclic stretch, inducing differentiation, and cytokine stimulation. B) Loading of bioactive substances. Engineered EVs can obtain desired cargos by endogenous loading (modifying donor cells) and exogenous loading (modifying EVs). Endogenous loading: Donor cells were transfected with plasmid, lentivirus, or Cas13d/crRNA. EVs secreting donor cells expressed specific proteins or gene editing tools. Exogenous loading: EVs were directly loaded with various therapeutic drugs (small‐molecule compounds, nucleotides) through sonication, electroporation, transfection, incubation, and extrusion. C) Targeted modification of EVs. To enhance the targeting efficacy of EVs, EVs were modified with exogenous peptides, proteins, or lipids by gene engineering (endogenous modification) and chemical modifications (exogenous modification). Endogenous modification: Donor cells were transfected with protein‐carried targeted peptides or exogenous protein plasmids. Exogenous modification: EV surfaces were directly conjugated with lipids which were incorporated into the membranes of EVs, or reacted with functional groups. D) Combined with nanomaterials. Nanomaterials, including liposomes and nanoparticles, can be mixed with EVs to form engineered EVs to enhance biological activity.

Preclinical and clinical studies have shown promising potential for EVs derived from natural cells in treating ALI/ARDS. However, natural EV therapy has some limitations. First, EVs are heterogeneous; the active substances they carry can vary with the condition of the donor cells, making it challenging to ensure consistency from different cell batches of EVs. Additionally, numerous distribution experiments have revealed that EVs are rapidly cleared in animals (half‐life < 10 min), limiting their sustained effects on target organs.^[^ ^16^ ^]^ These issues can be addressed using biotechnological approaches to modify and enhance EVs. In recent years, various biotechnologies have emerged to engineer EVs with enhanced tissue specificity, drug delivery efficiency, and biological activity.^[^ ^113^ ^]^ This section summarizes engineered strategies for enhancing the biological activity of EVs and their specific applications in treating ALI/ARDS (**Figure** **4**, **Table** **3**).

**Table 3:** Engineered EVs in ALI/ARDS.

Engineeringa)	Sources	Methodology	Animal model	Therapeutic outcomes	References
Alter the culture conditions of donor cells	rMSCs	Hypoxic preconditioning	LPS‐induced ALI model	Protected the integrity of epithelial barrier	[114]
	ECs	ECs subjected to cyclic stretching	Ventilator‐induced lung injury model	Promoted M2 macrophage polarization	[119]
	Raw264.7	M0 induced differentiation into M2	CLP‐induced sepsis model	Inhibited PMN migration and NET formation	[122]
	hMSCs	Cultured with a differentiation medium	LPS‐induced ALI model	Exhibited anti‐inflammatory and anticoagulant activities	[115]
	hMSCs	MSCs seeded on engineered hydrogels that mimic the elasticity of soft tissues	LPS‐induced ALI model	Secreted ≈10‐fold more EVs per cell than MSCs seeded on a rigid plastic substrate	[120]
	rMSCs	MSCs preconditioned with IFN‐γ	*E. coli*‐induced ALI model	Enhanced macrophage bacterial phagocytosis and killing activity	[116]
	mMSCs	MSCs preconditioned with IL‐1β	Sepsis‐induced lung injury model	Promoted endothelial cell repair and reduced inflammation and endoplasmic reticulum stress response	[117]
	hMSCs	MSCs preconditioned with TNF and IFN‐γ	LPS‐induced ALI model	Promoted M2 macrophage polarization	[125]
EVs as delivery vehicles for therapeutic drugs	rPMVECs	Syndecan‐1 overexpression	LPS‐induced ALI model	Ameliorated lung edema and inflammation and repaired the endothelial barrier	[128]
	rPMVECs	p18 overexpression	*P. aeruginosa*‐induced ARDS model	Reduced endothelial barrier permeability	[129]
	Dermal fibroblasts	Transduced with lentiviruses containing transgenes for ctnnb1 and gata4	Bleomycin‐induced lung injury model	Exhibited anti‐inflammatory and antifibrotic activity	[130]
	hMSCs	Nrf2 overexpression	LPS‐induced ALI model	Inhibited the activation of the NLRP3 inflammasome and promoted the polarization of M2 macrophages	[183]
	BEAS‐2B	CC16 overexpression	LPS‐ or bacteria‐induced ALI model	Inhibited the inflammatory and DNA damage responses	[184]
	mMSCs	HSF1 overexpression	Hemorrhagic shock‐induced lung injury model	Inhibited inflammation, reduced oxidative stress, decreased apoptosis, and protected the epithelial barrier.	[185]
	Dermal fibroblasts	IL‐10 or IL‐4 overexpression	LPS‐induced ARDS model	Reduced inflammation	[151]
	HEK293T	CD24 overexpression	LPS‐induced ARDS model	Modulated the host response to DAMPs without interfering with PAMPs’ immune recognition	[95]
	mMSCs	PD‐L1 overexpression	LPS‐induced pneumonia and ARSD model	Exerted immunosuppressive effects via the PD‐1/PD‐L1 signaling pathway	[186]
	HEK293T	Wnt3a overexpression	Elastase‐induced emphysema model	Activated Wnt signaling pathway and promoted cell growth	[131]
	HEK293T	Established CasRx/gRNA system	LPS‐induced ALI and septicemia model	Suppressed the proinflammatory response and ameliorated the damage	[133]
	MSCs	let‐7a‐5p overexpression	Hyperoxia‐induced lung injury model	Reduced macrophage infiltration and collagen deposition	[187]
	HL60 cells	Loaded with piceatannol via the pH gradient	LPS‐induced ALI and sepsis model	Reduced lung inflammation and lung edema	[135]
	HEK293T	Loaded with curcumin via incubation	LPS‐induced ALI model	Reduced inflammation	[136]
	Platelet	Loaded with dexamethasone via hydrophobic interaction	LPS‐induced ALI model	Downregulated the adaptive and innate immune responses, and alleviated the inflammation	[142]
	RAW264.7	Loaded with resveratrol and celastrol via sonication	LPS‐induced mouse sepsis model	Attenuated cytokine storm, reduced ALI, and increased survival of septic mice	[137]
	Platelets	Platelets were co‐incubated with TPAC‐1	LPS‐induced ALI model	Inhibited the infiltration of pulmonary inflammatory cells and calmed local cytokine storm	[139]
	iPSCs	Loaded with siRNA (against ICAM‐1) via electroporation	–	Inhibited the ICAM‐1 protein expression and PMN‐EC adhesion	[143]
	Serum	Loaded with siRNA (against Myd88) via electroporation	LPS‐induced ALI model	Decreased the secretion of cytokines and chemokines and the cellular infiltration	[94]
	Panax ginseng root	Loaded with miR‐182‐5p via electroporation	LPS‐induced ALI and sepsis model	Inhibited inflammation and reduced oxidative stress by targeting NOX4/Drp‐1/NLRP3 signal pathway	[147]
	HUVECs	Loaded with miR‐125b via ExoFect	LPS‐induced ALI model	Protected the integrity of endothelial barrier	[146]
	Serum	Loaded with ASO via electroporation	LPS‐ or *Klebsiella pneumonia*‐ or *E. coli*‐induced ALI model	Reduced the severity of lung injury and infiltration of neutrophil and reduced inflammatory cytokines and chemokines	[149]
	Red blood cell	Loaded with ASOs	SARS‐Cov‐2‐infected animal model	Suppressed SARS‐CoV‐2 replication	[80]
Targeted modification of EVs	HEK293T	Transfected with pDual‐ACE2 lentiviruses	SARS‐CoV‐2‐infected animal model	Blocked the binding of the viral spike protein RBD and prevented infections by SARS‐CoV‐2	[154]
	HUVECs	Transfected with LAMP‐2B‐LET lentiviruses	LPS‐induced ALI model	Prolonged retention time in lung tissues and targeted lung microvascular endothelial cells	[146]
	Dermal fibroblasts	SP‐A overexpression	LPS‐induced ARDS model	Promoted intrapulmonary retention	[151]
	HEK293T	Transfected with RBD‐VSVG vector	Transgenic mice that express human ACE2	Accumulated specifically in the target tissues that highly express ACE2	[152]
	HEK293T	Transfected with RBP‐LAMP‐2B expression vector	LPS‐induced ALI model	Interacted with RAGE and increased the intracellular delivery efficiency of curcumin	[136]
	Salmonella typhimurium	Decorated with RBD via SpyTag‐SpyCatcher	The golden Syrian hamster model of COVID‐19	Produced high titers of blood anti‐RBD IgG and detectable mucosal reactions	[156]
	Lung spheroid cells	Decorated with RBD via chemical conjugation	SARS‐CoV‐2‐infected animal model	Elicited RBD‐specific IgG antibodies, mucosal IgA responses, and CD4^+^ and CD8^+^ T cells with a Th1‐like cytokine expression profile in the lung tissues and cleared SARS‐CoV‐2 pseudovirus after a challenge	[155]
	RAW264.7	Modified with FA by chemical conjugation	LPS‐induced sepsis model	Improved the accumulation in the lung tissue and targeted inflammatory macrophages	[137]
	Panax ginseng root	Coated with the neutrophil membrane via incubation	LPS‐induced ALI and sepsis model	Enhanced targeting of lung tissue	[147]
Combined with nanomaterials	HEK293T	ACE2 overexpression HEK293T cells incubate with PDA nanoparticles	S protein‐induced inflammation model	Competed with ACE2‐expressing ECs for S protein binding; Attenuated the level of inflammatory cytokines by mediating oxidative stress	[157]
	Platelet and L929 cells	PLGA cloaked with platelet‐derived EVs and the calreticulin‐expressed membrane	LPS‐induced ALI model	PC@PLGA specifically targeted activated neutrophils and misled macrophages to recognize them as “aged” neutrophils and then initiated premature PrCR and prevented proinflammatory response and tissue damage	[158]
	Serum	Constructed a serum exosomal and liposomal hybrid nanocarrier which modified with D Nase I‐MMP‐9 and encapsulated with MPS.	LPS‐induced ALI model	Inhibited the coordinated action of neutrophils and macrophages and remodeled lung immune homeostasis	[159]
	MSCs	EV preincubated with HA	*P. aeruginosa*‐induced pneumonia	Increased the potency of MSCs‐V in PA pneumonia in part by enhancing the trafficking of MSCs‐EV to the sites of inflammation	[163]

a) BEAS‐2B: human normal lung epithelial cells; RAW264.7: mouse mononuclear macrophages cells; HUVECs: human umbilical vein endothelial cells; CC16: Club Cell Protein 16; “h” stands for human, “r” for rat and “m” for mouse.

### 4.1 Altering the Culture Conditions of Donor Cells

The biological activity of EVs can be altered by intentionally adjusting the culture conditions of donor cells. This can be achieved by introducing specific stimuli,^[^ ^114^ ^]^ inducing cell differentiation,^[^ ^115^ ^]^ or adding cytokines during cell culture.^[^ ^116^, ^117^ ^]^

Undifferentiated MSCs primarily rely on glycolytic metabolism for energy, so hypoxia preconditioning can boost their multipotency and proliferation.^[^ ^118^ ^]^ Ren et al.^[^ ^114^ ^]^ cultured MSCs under hypoxic conditions to derive hypoxia‐induced MSC‐EVs or hypo‐EVs. These hypo‐EVs contained higher levels of lncRNA XIST, which served as an miR‐455‐3p sponge targeting Claudin‐4 in ECs, thereby maintaining the integrity of EC junctions. In another study, Wang et al.^[^ ^119^ ^]^ reported that EVs released by ECs subjected to cyclic stretching promoted the polarization of macrophages to an anti‐inflammatory phenotype in ventilator‐induced lung injury. At the molecular level, cyclic stretching stimulated the upregulation of miRNA‐21a‐5p in EVs, inhibiting the expression of Notch2 and SOCS1. Combining EVs with matrix materials can enhance their production without altering their biological activities. Lenzini et al.^[^ ^120^ ^]^ developed a hydrogel substrate composed of alginate polymer combined with the cell adhesion peptide Arg‐Gly‐Asp (RGD). MSCs cultured on this hydrogel substrate secreted more EVs compared with those seeded on rigid plastic substrates, without affecting their therapeutic efficacy in a mouse model of ALI.

**Figure 5:**
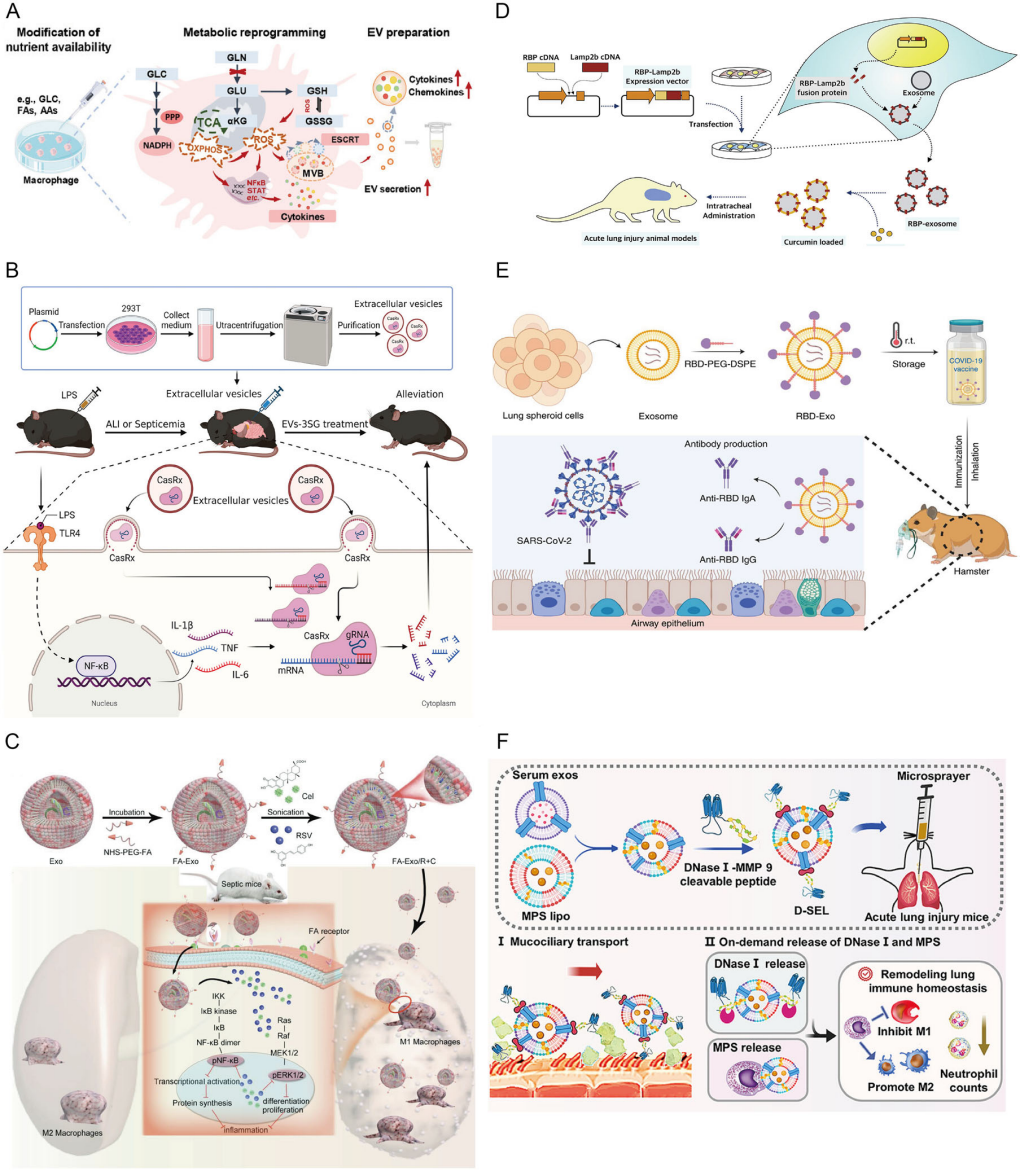
Application of engineered EVs of different modification strategies in the treatment of ALI/ARDS and related lung diseases. A) Altering the culture medium to induce specific differentiation in donor cells can enhance EV bioactivity. GLN−depletion efficiently enhances EV biogenesis and cargo packaging. Adapted with permission.^[^ ^121^ ^]^ Copyright 2024, the authors. B) Endogenous loading of EVs. The engineered EV‐delivered CasRx/gRNA system degrades TNF, IL‐1β, and IL‐6 mRNAs to relieve the cytokine storm triggered by LPS, alleviating acute inflammatory diseases in mice. Adapted with permission.^[^ ^133^ ^]^ Copyright 2023, the authors. C) Exogenous loading of EVs. Macrophage‐derived Exo was incubated with NHS‐PEG‐FA vector to form FA‐Exo, and then the RSV and Cel mixture was loaded into FA‐Exo by sonication to obtain FA‐Exo/R + C, which exhibits strong anti‐inflammatory and immunosuppressive activities. Adapted with permission.^[^ ^137^ ^]^ Copyright 2023, Wiley‐VCH GmbH. D) Targeted modification of EVs through gene engineering. RBP‐Evs, created by genetic modification technology to fuse the RBP with the membrane protein Lamp‐2B on EVs, alleviate acute inflammatory diseases in ALI animal models. Adapted with permission.^[^ ^136^ ^]^ Copyright 2020, Elsevier B.V. E) Targeted modification of Evs through chemical modification. RBD‐EVs are obtained by modifying the RBD in DSPE‐PEG‐NHS to obtain DSPE‐PEG‐RBD and then inserting DSPE‐PEG‐RBD into the membrane surface of EVs via hydrophobic interactions, which has been regarded as a promising inhalable COVID‐19 vaccine to alleviate acute inflammatory. Adapted with permission.^[^ ^155^ ^]^ Copyright 2022, the authors. F) Combining EVs with nanomaterials to improve drug delivery efficiency. A hybrid nanoplatform (D‐SEL), shaped by conjugating DNase I to a serum exosomal and liposomal hybrid nanocarrier (SEL) via a matrix metalloproteinase 9 (MMP‐9)‐cleavable peptide, and has been encapsulated with MPS, remodels lung immune homeostasis in ALI. Adapted with permission.^[^ ^159^ ^]^ Copyright 2023, American Chemical Society.

Altering the culture medium to induce specific differentiation in donor cells can enhance EV bioactivity^[^ ^121^ ^]^ (**Figure** **5**A). Kaspi et al.^[^ ^115^ ^]^ induced MSCs to differentiate into neurotrophic and immunomodulatory factor‐secreting cells, termed MSC‐NTFs. EVs derived from these MSC‐NTFs elevated the levels of growth factors, which significantly inhibited inflammatory and coagulation responses in an ARDS animal model. Similarly, Jiao et al.^[^ ^122^ ^]^ polarized macrophages into M2‐type macrophages to obtain M2 macrophage‐derived EVs by adding IL‐4 and IL‐13 to the culture medium. Compared with undifferentiated macrophage‐derived EVs, M2‐EVs were enriched with prostaglandin E2 (PGE2), which increased 15‐LO expression, shifted lipid mediator biosynthesis from LTB4 to LXA4 in polymorphonuclear neutrophils, inhibited neutrophil migration and extracellular trap formation, and ultimately reduced lung injury.

Under the stimulation of various proinflammatory cytokines, MSCs exhibit immunosuppressive properties, enhancing their immune regulatory ability and improving their therapeutic effect in various inflammatory diseases.^[^ ^123^, ^124^ ^]^ The EVs exhibit similar properties. Wei‐Ting Lin et al.^[^ ^125^ ^]^ pretreated MSCs with TNF and IFN‐γ, resulting in IFN‐γ/TNF‐MSCs‐EVs, which showed anti‐inflammatory effects in LPS‐induced ALI models. Similarly, Varkouhi et al.^[^ ^116^ ^]^ utilized IFN‐γ to stimulate MSCs, producing IFN‐γ‐MSC‐EVs. In bacterial‐induced lung injury models, IFN‐γ‐MSC‐EVs effectively enhance phagocytosis and killing activity of macrophages against bacteria. Cheng et al.^[^ ^117^ ^]^ pretreated MSCs with IL‐1β to obtain IB‐EVs, which inhibited endoplasmic reticulum stress and promoted injury repair in sepsis‐induced lung injury by increasing SIRT1 expression and ERK phosphorylation.

However, adjusting the donor cells’ culture conditions risks altering EVs’ biological activity. lncRNA‐p21 expression in EVs produced by LPS‐treated MSCs significantly increases, but excessive LPS stimulation may lead to cellular stress responses, affecting the quality and stability of EVs. Definitive research conclusions are lacking on whether MSC‐derived exosomes produced under LPS or other proinflammatory cytokine exposure conditions affect ECs during the progression of ALI. Additionally, adjusting culture conditions may lead to an increase in the heterogeneity of EVs. Different cellular states and stress responses may produce different types of EVs, which may vary in size, morphology, and composition, affecting their stability and consistency in clinical applications.

### 4.2 Loading of Bioactive Substances/EVs as Delivery Vehicles for Therapeutic Drugs

#### 4.2.1 Modifying Donor Cells/Endogenous Loading

The bioactive substances carried by EVs can be modified by altering the donor cells through gene editing. By performing specific gene editing on donor cells, the biological activity of EVs can be altered. Utilizing genetic engineering techniques to construct a target protein expression vector and introducing it into donor cells, the EVs produced by these cells can carry relevant target proteins to enhance their biological effects.

Syndecan‐1, an endothelial barrier protective protein,^[^ ^126^, ^127^ ^]^ was targeted in a study by Zhang et al.^[^ ^128^ ^]^ They constructed a Syndecan‐1 overexpression lentiviral vector to transfect mouse lung microvascular endothelial cells, producing high Syndecan‐1 expression EVs (SDC1‐high‐EVs). In ALI models, these SDC1‐high‐EVs significantly alleviated lung edema, reduced inflammatory responses, and restored endothelial barrier function. p18 is an endosomal protein that participates in reducing pulmonary endothelial permeability in ARDS. Harrington et al.^[^ ^129^ ^]^ constructed a p18 overexpression plasmid to transfect lung microvascular endothelial cells, obtaining p18‐overexpressing EVs. In animal models of ARDS, p18‐EVs enhance endothelial barrier integrity by upregulating barrier‐protective miRNAs. Ibrahim et al.^[^ ^130^ ^]^ used dermal fibroblasts transfected with CTNNB1 and GATA4 to create specialized activated tissue effector EVs (ASTEX). ASTEX demonstrated anti‐inflammatory and antifibrotic effects in bleomycin‐induced ALI models, thereby preventing the progression of ALI to pulmonary fibrosis. Wnt3a, a hydrophobic protein, is involved in tissue regeneration in chronic obstructive pulmonary disease. Gao et al.^[^ ^131^ ^]^ engineered Wnt3a^WG^EVs by coexpressing Wnt3a with the membrane protein WLS and an engineered glypican GPC6^ΔGPI^‐C1C2. Wnt3a^WG^EVs activate the Wnt signaling pathway, promoting the growth of damaged alveolar ECs.

EVs can also serve as delivery vehicles for gene editing tools. CRISPR/CasRx (Cas13d) is an important gene‐editing tool that transiently affects the transcriptome of genes by targeting and cleaving specific RNA sequences.^[^ ^132^ ^]^ Li et al.^[^ ^133^ ^]^ developed engineered EVs to deliver CasRx/gRNA complexes to treat ALI. The tPA was selected as the signaling peptide, and plasmids expressing CasRx and the gRNAs for three inflammatory cytokines, TNF, IL‐6, and IL‐1β, were used to transfect donor cells. The released EVs were loaded with CasRx/gRNA gene editing tools. In an LPS‐induced ALI animal model, EVs‐CasRx/gRNA effectively reduced the levels of TNF, IL‐6, and IL‐1β. This intervention resulted in reduced alveolar wall thickening, lung edema, and collagen deposition, while improving lung tissue structure. It played an important therapeutic and reparative role in ALI (Figure 5B).

Utilizing genetic engineering techniques, target protein expression vectors are constructed and introduced into donor cells. The CRISPR/CasRx (Cas13d) gene editing tool is employed to transiently affect the transcriptome by targeting and cleaving specific RNA sequences. Both approaches enable cells to produce EVs carrying the relevant target proteins, significantly enhancing therapeutic efficacy, which is a widely applied engineering technology at present.

Nevertheless, genetic engineering operations may introduce potential safety risks. Transfection with viral vectors can integrate into the host cell genome, leading to gene mutations or insertional mutations, which may cause adverse outcomes such as cellular carcinogenesis. Gene editing technologies also have off‐target effects, which may damage nontarget genes and affect the normal physiological functions of cells. Moreover, the preparation process for genetically engineered EVs is complex, and quality control is challenging, making large‐scale production and application difficult, thus limiting the promotion of their clinical use.

#### 4.2.2 Modifying EVs/Exogenous Loading

EVs, with their lipid bilayer structure, high biocompatibility, low toxicity, and small size, make for ideal natural nanocarriers.^[^ ^134^ ^]^ They can be directly loaded with various therapeutic drugs (small‐molecule compounds and nucleotides) through chemical or physical means.

##### Small Molecule Compounds

The utilization of EVs for delivering small‐molecule drugs significantly enhances drug bioavailability, stability, and accumulation in target cells. Gao et al.^[^ ^135^ ^]^ developed neutrophil‐derived engineered EVs loaded with the anti‐inflammatory drug piceatannol using a pH gradient, which effectively alleviated LPS‐induced ALI. Kim et al.^[^ ^136^ ^]^ loaded curcumin, a highly hydrophobic drug with anti‐inflammatory activity, into EVs through coincubation, which significantly reduced the levels of proinflammatory factors in animal models of ALI.

Some target‐modified EVs can improve drug delivery efficiency. Zheng et al.^[^ ^137^ ^]^ utilized folic acid‐functionalized EVs (FA‐EVs) targeting inflammatory areas loaded with the anti‐inflammatory drug resveratrol and the immunosuppressive drug celastrol (FA‐EVs/R + C). In the LPS‐induced sepsis model, FA‐EVs/R + C exhibited strong anti‐inflammatory and immunosuppressive activities, reduced cytokine storms and ALI, and improved the survival rate of mice with sepsis (Figure 5C).

With the assistance of specific proteins expressed on the surface, platelet‐derived EVs (P‐EVs) accumulate at inflammatory sites without aggravating inflammation.^[^ ^138^ ^]^ Ma et al.^[^ ^139^ ^]^ used P‐EVs loaded with the inflammatory factor inhibitor TPCA‐1, which significantly reduced the inflammatory response and prevented cytokine storms compared to TCPA‐1 alone.^[^ ^140^ ^]^

Dexamethasone (DEX) is an anti‐inflammatory steroid that suppresses the inflammatory response and induces several side effects during long‐term use.^[^ ^141^ ^]^ Based on its hydrophobic properties, Ma et al.^[^ ^142^ ^]^ loaded DEX into P‐EVs via coincubation methods, specifically targeting inflamed lung tissue. Compared with the DEX treatment group, EVs loaded with DEX reduced the required dosage, effectively avoiding the occurrence of side effects.

The use of engineered EVs for the delivery of small‐molecule drugs can significantly enhance the bioavailability, stability, and accumulation of drugs in target cells. Some EVs with targeting modifications can also improve the efficiency of drug administration. However, when used alone, this engineering technology still has limitations. The most obvious are the limitations in drug loading efficiency and targeting accuracy in drug delivery.

The interaction between drug molecules and the membrane may not be entirely ideal, and some drugs may not be able to effectively enter the EVs, leading to insufficient drug loading each time and affecting the therapeutic effect. Moreover, the chemical properties of different drug molecules vary greatly. Some hydrophobic drugs may be easier to load into exosomes, while hydrophilic drugs may require more complex loading methods, increasing the difficulty and uncertainty of the loading process.

Additionally, the targeting modification of engineered exosomes is mainly based on specific receptor‐ligand interactions. In the complex physiological environment in vivo, the targeting ligands may bind to similar receptors on nontarget cells, leading to nonspecific drug delivery and reducing targeting accuracy.

##### Nucleotides

Small interfering RNA (siRNA), a 20–25 nucleotide double‐stranded RNA, serves as a crucial gene therapy agent by interfering with the expression of specific genes. EVs are widely regarded as ideal siRNA delivery vehicles due to their nonimmunogenicity, biodegradability, and excellent biocompatibility. Ju et al.^[^ ^143^ ^]^ engineered EVs with ICAM‐1‐siRNA via electroporation. In lung microvascular endothelial cells, these engineered EVs can directly inhibit the expression of ICAM‐1 and reduce the adhesion between neutrophils and endothelial cells, thereby suppressing inflammation. To boost siRNA delivery to lung tissue, Han et al.^[^ ^94^ ^]^ packaged Myd88‐siRNA into EVs and delivered them to damaged lung tissues using a vibrating mesh nebulizer. Studies have shown that siRNA encapsulated in EVs can effectively reduce degradation and enzymatic digestion in vivo and enhance its action in target cells. In ALI animal models, these engineered EVs reduced proinflammatory factors and chemokines, inflammatory cell infiltration, and pulmonary edema.

miRNAs are endogenous, single‐stranded RNA fragments, typically 21–23 nucleotides long, that regulate gene expression by interacting with the 3'UTR of mRNA.^[^ ^144^ ^]^ EVs serve as important delivery vectors for miRNAs.^[^ ^145^ ^]^ MiRNA‐125b exhibits anti‐inflammatory and vascular repair properties. Gu et al.^[^ ^146^ ^]^ loaded miRNA‐125b into EVs using an ExoFect transfection kit. These engineered EVs delivered miRNA‐125b to injured endothelial cells, inhibiting apoptosis, promoting proliferation, restoring intercellular connections, and protecting the endothelial barrier integrity. Similarly, Ma et al.^[^ ^147^ ^]^ used electroporation to load miRNA‐182‐5p into ginseng root‐derived ELNs. These engineered EVs improved ALI in sepsis by inhibiting the target genes NOX4 and the Nox4/Drp‐1/NLR3 signaling pathway.

Antisense oligonucleotides (ASOs) are small DNA sequences that reduce gene expression at the post‐transcriptional level.^[^ ^148^ ^]^ EVs can enhance their bioactivity by loading ASO. Han et al.^[^ ^149^ ^]^ engineered EVs loaded with ASO via electroporation, effectively reducing lung inflammation in ALI mice by knocking down Lncenc1 expression. Jayasinghe et al.^[^ ^80^ ^]^ utilized red blood cell‐derived EVs to load ASO targeting a key conserved region of SARS‐CoV‐2. These ASO‐loaded RBC‐EVs were efficiently absorbed by cells and inhibited SARS‐CoV‐2 replication in vitro and in vivo.

Small interfering RNA (siRNA), microRNA (miRNA), and others are susceptible to degradation by nucleases in vitro, which affects their stability and therapeutic efficacy. In contrast, EVs can provide a relatively stable internal environment for nucleic acid drugs, protecting them from degradation.

The loading process can be accomplished with the help of methods such as sonication, electroporation, and coincubation.^[^ ^150^ ^]^ However, it may face issues such as changes in the structure of EVs and low loading rates. Loading can also be carried out through genetic engineering approaches. Methods mentioned in the examples above allow for more precise regulation of miRNA secretion, but they require higher standards for the physiological state of cells and the technical skills of the operators.

### 4.3 Targeted Modification of EVs

To improve the therapeutic efficiency of EVs, their surfaces can be modified with specific target molecules such as peptides, proteins, and antibodies to enhance lung targeting and reduce hepatic clearance. Chemical modifications and gene engineering are the two primary methods commonly employed for these modifications.

#### 4.3.1 Gene Engineering

Targeted peptides are polypeptides with specific targeting ability. Due to their low molecular weight, they are often fused with transmembrane proteins expressed on EVs, such as CD63 and Lamp2b, to create expression vectors. After transfecting donor cells, the resulting EVs express the targeted peptide on their surface, thus acquiring targeting capabilities. Kim et al.^[^ ^136^ ^]^ utilized genetic modification technology to fuse the receptor for advanced glycation end‐product receptor (RAGE)‐binding peptide (RBP) with the membrane protein Lamp‐2B on EVs, creating RBP‐EVs (Figure 5D). These RBP‐EVs colocalize with type I alveolar ECs. In the LPS‐induced ALI model, RBP‐EVs can efficiently deliver the anti‐inflammatory drug curcumin to the injured lungs, compared with unmodified EVs, enhancing an anti‐inflammatory response. Similarly, Gu et al.^[^ ^146^ ^]^ modified the lung microvascular endothelial cell‐targeting peptide (LET) on the surface of EVs using genetic engineering. These modified EVs exhibited significantly prolonged retention in the lung tissue, directly targeting injured lung microvascular endothelial cells and promoting repair of the endothelial barrier.

Gene engineering can be utilized to overexpress target proteins and extend their retention in the lung tissue through receptor‐ligand interactions. For example, SP‐A, an alveolar surfactant protein, binds to various receptors in the lung tissue to prolong retention time. Salazar‐Puerta et al.^[^ ^151^ ^]^ engineered EVs to express SP‐A, resulting in increased retention and accumulation in lung tissue, which enhanced drug delivery and therapeutic efficacy. In COVID‐19 research, Fu et al.^[^ ^152^ ^]^ modified the receptor‐binding domain of SARS‐CoV‐2 spike protein on EV membranes, encapsulating siRNAs targeting the virus. These engineered EVs specifically targeted damaged lung tissues, delivering siRNAs that exerted antiviral effects.

Furthermore, targeted protein‐modified EVs can serve as important neutralizing therapeutic agents. During the COVID‐19 pandemic, SARS‐CoV‐2 primarily infects host cells by binding to angiotensin‐converting enzyme 2 (ACE2) receptors.^[^ ^153^ ^]^ Blocking this interaction is an important preventive measure. El‐Shennawy et al.^[^ ^154^ ^]^ used genetic engineering to create EVs expressing ACE2 (evACE2). Compared with the recombinant human ACE2 protein, evACE2 can efficiently disrupt the binding between the virus and host, effectively preventing SARS‐CoV‐2 infection. In hACE2 transgenic mice, evACE2 effectively protected the mice against lung injury and death caused by SARS‐CoV‐2.

#### 4.3.2 Chemical Modification

EVs are nonbiological entities with lipid bilayer structures that allow for direct chemical modification of their membrane surfaces.

Target molecules can modify the surface of the EVs by binding to lipid materials under hydrophobic forces. For example, Wang et al.^[^ ^155^ ^]^ modified the RBD in DSPE‐PEG‐NHS to obtain DSPE‐PEG‐RBD and then inserted DSPE‐PEG‐RBD into the membrane surface of EVs via hydrophobic interactions to obtain RBD‐EVs (Figure 5E). These RBD‐EVs serve as important COVID‐19 vaccines by triggering humoral and cellular immune responses to combat SARS‐CoV‐2. Furthermore, targeting molecules react with surface functional groups (e.g., carboxyl and amine groups) to enhance their targeting abilities. Zheng et al.^[^ ^137^ ^]^ combined an inflammation‐targeting FA with NHS‐PEG to create NHS‐PEG‐FA. This compound anchors FA to the surface of EVs via an amide reaction with primary amines on the surface of EVs. In animal models of LPS‐induced ALI, FA‐EVs accumulated in the lungs after systemic administration, demonstrating the potential for targeted delivery to activated macrophages. Jiang et al.^[^ ^156^ ^]^ utilized SpyTag‐SpyCatcher protein conjugation technology to modify EV surfaces with the RBD, creating an RBD‐OMV vaccine. Intranasal administration of this vaccine in Syrian hamsters with COVID‐19 induced the production of anti‐RBD IgG.

Notably, both genetic engineering and chemical modification may face issues such as increased immunogenicity and reduced biocompatibility. These risks need to be excluded in the actual application process.

### 4.4 Combining EVs with Nanomaterials

EVs, known for their superior biocompatibility and specific protein‐loading capacity, can be combined with various nanomaterials to improve drug delivery efficiency.

During the COVID‐19 pandemic, Ma et al.^[^ ^157^ ^]^ developed a Nano‐Bait (PDA@Exosome) to inhibit SARS‐CoV‐2 infection. PDA@Exosomes were obtained by coculturing 293 T cells overexpressing ACE2 with polydopamine (PDA) nanoparticles. In lung injury models, PDA@Exosomes competed with ACE2‐expressing ECs for S protein binding, thereby reducing lung damage. Moreover, due to the ability of PDA to scavenge free radicals, PDA@Exosomes also reduced the production of inflammatory factors by mediating oxidative stress.

Chen et al.^[^ ^158^ ^]^ used P‐EVs and cell membranes expressing calreticulin (CRT) to cloak poly (lactic‐co‐glycolic acid) (PLGA) to obtain PC@PLGA. In an ALI mouse model, PC@PLGA specifically targeted activated neutrophils through P‐selectin derived from P‐EVs and initiated macrophage‐mediated programmed cell clearance through CRT, effectively eliminating activated neutrophils and preventing inflammatory responses.

Liu et al.^[^ ^159^ ^]^ designed a hybrid nanoplatform (D‐SEL) by conjugating DNase I as an outer cleavable arm to a serum exosomal and liposomal hybrid nanocarrier (SEL) via a matrix metalloproteinase 9 (MMP‐9)‐cleavable peptide and encapsulated it with methylprednisolone sodium succinate (MPS) (Figure 5F). In ALI animal models, D‐SEL promotes the polarization of macrophages toward an anti‐inflammatory phenotype, degrades neutrophil extracellular traps (NETs), disrupts the synergistic interaction between neutrophils and macrophages, and reshapes the pulmonary immune microenvironment through a sequential drug delivery mechanism.

Holay et al.^[^ ^160^ ^]^ encapsulated DEX‐loaded PLGA nanoparticles (DEX‐NPs) in EVs using ultrasonic methods to obtain biomimetic nanoparticles (DEX‐EV‐NPs).^[^ ^161^ ^]^ After intravenous administration, DEX‐EV‐NPs rapidly accumulated in the lungs, effectively reducing the level of the proinflammatory factor IL‐6 in the lungs while also reducing the toxicity of DEX. No significant changes were observed in major organs or hemograms.

Hyaluronic acid (HA),^[^ ^162^ ^]^ a nonsulfated glycosaminoglycan, known for its high molecular weight (HMW) HA (≥1.0 MDa), can enhance the secretion of anti‐inflammatory cytokines by target cells, maintaining the integrity of endothelial and epithelial barriers and promoting tissue regeneration and repair. Zhou et al.^[^ ^163^ ^]^ pretreated MSC EVs with HMW HA, which significantly increased the efficacy of MSC EVs against PA. HMW HA‐induced MSC‐EVs further increased monocyte phagocytosis and bacterial clearance compared with the MSC‐EV group.

This engineering technology is not yet mature enough. For example, the in vivo dynamic behavior and targeting of hybrid nanocarriers mentioned earlier may be affected by the respective characteristics of EVs and liposomes, which need to be further optimized. The high preparation cost also limits its promotion in clinical applications.

In fact, no matter what kind of engineering method has its unique advantages and limitations, the combination of different methods may get better results. Researchers should choose the approach that best meets their needs according to the specific research objectives and experimental conditions. But at the same time, the preparation cost is more complex, so that production standardization and clinical transformation may be more difficult.

## 5 Challenges and Perspectives

Over the past decade, scientists have explored EVs extensively to understand their mechanisms of action better. EVs exhibit good biocompatibility and potent bioactivity in the treatment of lung diseases and hold great potential for clinical applications. Compared with ordinary cell therapy, EVs have high stability, low immunogenicity, and excellent biocompatibility. EVs possess a lipid bilayer structure that serves as a natural nanomaterial. Faced with traditional chemically synthesized nanomaterials, EVs exhibit superior biocompatibility and low toxicity. Particularly, compared with the traditional treatments for ALI/ARDS mentioned earlier, EVs can obviously exert a more proactive therapeutic effect by carrying various bioactive molecules, directly penetrating the air‐blood barrier, and participating in the processes of injury repair. Significant progress has been made in preclinical and clinical studies using EVs of various origins to treat ALI/ARDS.

Nevertheless, natural EV therapy faces limitations due to heterogeneity and a short half‐life, which affect therapeutic efficiency and targeting ability. Notably, certain risks remain associated with EV use in therapy. For instance, while MSC‐derived EVs exhibit low immunogenicity, they may still trigger severe immune responses in some instances, particularly with repeated administrations or high dosages. This could lead to inflammatory reactions and tissue damage, potentially affecting cell proliferation and differentiation, thereby increasing the risk of tumor formation.^[^ ^14^ ^]^ To enhance the biological activity of EVs in preclinical studies, EVs have been engineered and modified to improve their targeting and efficiency.

Therapeutic approaches based on EVs have tremendous potential in alleviating lung injury to prevent ALI/ARDS. However, many key issues must still be addressed for clinical translation.

Firstly, optimizing the production process and enhancing the production efficiency for EVs are critical issues that must be addressed urgently. In clinical trials of EV treatment for ARDS, the administered dose of EVs is generally high (0.9–1.2 × 10^12^ particles per dose for intravenous infusion). The traditional preparation process for EVs is slow, expensive, and inefficient, limiting the demand for large‐scale clinical applications. Consequently, enhancing the yield of EVs has become a crucial issue that requires urgent attention. Various physical, biological, and chemical stimulation methods have been employed to increase EV yield.^[^ ^164^ ^]^ Hao et al.^[^ ^165^ ^]^ developed a microfluidic editing platform that can squeeze and stimulate cells in a high‐throughput and noninvasive manner to enhance EV secretion. Watson et al.^[^ ^166^ ^]^ employed a hollow‐fiber bioreactor to culture cells, enabling the efficient production of bioactive EVs in a short time, thus improving production efficiency.

**Figure 6:**
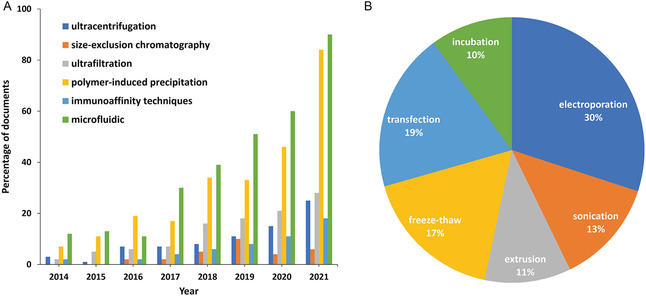
Statistics of reported exosome production processes. A) Trend of exosome isolation/purification methods during 2014–2021. B) Percentages of various exosome loading methods in therapy and diagnostics (from ref. [167] Copyright 2022, the authors).

EVs are a large collection of subsets including exosomes, microvesicles, and apoptotic bodies. The isolation and purification of EVs to obtain a single type are beneficial for standardization. Currently, methods for EV isolation and purification mainly include ultracentrifugation, ultrafiltration, immunoaffinity, polymer precipitation, microfluidics, and size‐exclusion chromatography. A previous study^[^ ^167^ ^]^ counted the exosome applications in therapeutics and diagnostics concerning various exosome isolation methods during the years 2014−2021 (**Figure** **6**A), which implied that microfluidics‐based isolation techniques and polymer‐based precipitation techniques were the most commonly used methods in recent years. They are rapid and simple but have existing problems of low sample capacity and low purity. The traditional ultracentrifugation is appropriate for large‐volume samples and cost‐effective, but it faces high equipment costs, low yield, and potential damage to EVs. According to different application purposes, different methods can be used for EV standardized production.

Secondly, due to the excellent cell communication ability, EVs have been regarded as an efficient drug delivery vehicle. Various biotechnological approaches have been employed to engineer and modify EVs, such as sonication, electroporation, transfection, incubation, and extrusion^[^ ^167^ ^]^ (Figure 6B), enhancing their biological activity. However, these modifications are double‐edged swords that may pose risks while improving the biological activity of EVs. For instance, some studies used lentiviral vectors as transfection tools to introduce functional proteins, miRNAs, and targeted peptide sequences into EVs. However, lentiviral vectors may pose the risk of integrating viral genomes into the host genome cell. Therefore, safer modification methods such as plasmid transfection and chemical modification are recommended for further research on engineered EVs. Additionally, EVs, as natural nanocarriers, can be loaded with small molecules or gene drugs using ultrasound and electroporation, but these methods may disrupt their original structure. Therefore, finding safer, more efficient, and gentler EV modification techniques has become an important topic in current research. Yang et al.^[^ ^168^ ^]^ developed a cellular‐nanoporation (CNP) biochip to produce large amounts of exosomes containing therapeutic mRNA and targeted peptides. Cells of various origins were transfected with plasmid DNA and stimulated with local and transient electrical stimulation to promote the release of exosomes carrying transcriptional mRNA and targeted peptides. Compared to other exosome production strategies, cellular nanopores produced up to 50 times as many exosomes and more than 103 times as many exosome mRNA transcripts, even from cells with low basal levels of exosome secretion, which may provide a solution for exosomes to effectively and stably load nucleic acid drugs.

In addition, the storage conditions of EVs are crucial factors that affect their yield and function. Currently, the commonly used storage method for EVs is at −80 °C. The addition of cryoprotectants (e.g., human albumin and trehalose) reduces the impact of freeze‐thaw processes and improves the stability of EVs during long‐term storage and freeze‐thaw cycles.^[^ ^169^ ^]^ Standardizing the preparation of natural and engineered EVs and controlling their quality are crucial for their therapeutic applications. The International Society for Extracellular Vesicles (ISEV) released guidelines for EV preparation in 2014,^[^ ^170^ ^]^ 2018,^[^ ^171^ ^]^ and 2023,^[^ ^12^ ^]^ further evaluating the standards of production quality of EVs. In subsequent clinical studies, it will be necessary to develop more efficient isolation and purification processes under GMP conditions. This will meet stringent regulatory requirements and ensure high purity and consistency across EV batches.

Notably, the classification of EVs is an important regulatory hurdle. EVs may be classified as biotechnology products or advanced therapy medicinal products (ATMP), which affects the scope of subsequent development studies and the approval process.^[^ ^172^ ^]^ Regardless of the registration procedure, the applicant must carefully define the scope of the development research based on the characteristics of the source cell, manufacturing process, and carrying molecules. Preclinical studies for extracellular vesicle drugs are very different from conventional chemical drugs. Focusing on biologically effective doses and safety is essential, along with obtaining reliable data through methods such as animal experiments and 3D culture. Overall, the complexity of the regulatory environment is a major obstacle to the commercialization of EVs. Close collaboration with regulatory authorities is essential to develop a sound development strategy based on product characteristics, ensuring quality, safety, and effectiveness, and ultimately securing marketing authorization.

Although EVs have demonstrated strong therapeutic effects in preclinical trials for ALI/ARDS, key factors need to be considered when transitioning EVs to clinical application, especially the administration route and dosing regimen of EVs. Current preclinical studies show significant variations in dosage and administration regimen owing to differences in EV isolation and preparation methods. The emergence of the COVID‐19 pandemic has accelerated the initiation and progression of numerous clinical trials employing EVs as potential therapeutic modalities for the treatment of ARDS. In these trials, EVs were quantified based on the number of particles. Consequently, it may be a better choice when studying dosage in future preclinical research. Moreover, defining robust potency assay methods for evaluating therapeutic efficacy is paramount to maximizing the effectiveness of these treatments and ensuring safety in the long therapeutic process.

Lastly, ethical guidelines for the clinical use of EVs are currently lacking. In the future, establishing and improving relevant ethical norms is essential to protect patients’ rights and promote the correct use of EVs.

## 6 Conclusion

Overall, natural EVs play significant roles in various physiological activities associated with ALI/ARDS, such as the regulation of inflammation, inhibition of apoptosis, promotion of barrier repair, and modulation of autophagy. Understanding their mechanisms, improving quality standards, and confirming optimal dosing and treatment regimens are essential for promoting the clinical use of EVs. Recent significant progress in research on engineered EVs for ALI treatment has indicated their potential as a new class of therapeutics offering good biocompatibility, high efficacy, and customization. Therefore, future endeavors should prioritize optimizing and stabilizing these engineered EVs to ensure their safety and effectiveness, while achieving scalable production for clinical applications.

## Conflict of Interest

The authors declare no conflict of interest.

## Author Contributions

**Zhengyan Gu**: investigation (equal); writing—original draft (lead); writing—review & editing (equal). **Wenjun Xue**: investigation (equal); writing—original draft (equal); writing—review & editing (lead). **Guanchao Mao**: investigation (equal); writing—original draft (equal); writing—review & editing (equal). **Zhipeng Pei**: investigation (equal); writing—original draft (equal); writing—review & editing (equal). **Jingjing Li**: investigation (supporting); writing—review & editing (supporting). **Mingxue Sun**: supervision (equal); writing—review & editing (equal). **Xinkang Zhang**: supervision (equal); writing—review & editing (supporting). **Shanshan Zhang**: supervision (equal); writing—review & editing (supporting). **Songling Li**: supervision (equal); writing—review & editing (supporting). **Jinfeng Cen**: investigation (supporting); writing—review & editing (supporting). **Kai Xiao**: conceptualization (equal); writing—review & editing (supporting). **Ying Lu**: conceptualization (equal); writing—review & editing (supporting). **Qingqiang Xu**: conceptualization (lead); writing—review & editing (supporting). **Zhengyan Gu**, **Wenjun Xue**, **Guanchao Mao**, and **Zhipeng Pei** contributed equally to this work.
